# Transcriptome and secretome analysis of *Aspergillus fumigatus* in the presence of sugarcane bagasse

**DOI:** 10.1186/s12864-018-4627-8

**Published:** 2018-04-03

**Authors:** Paula Fagundes de Gouvêa, Aline Vianna Bernardi, Luis Eduardo Gerolamo, Emerson de Souza Santos, Diego Mauricio Riaño-Pachón, Sergio Akira Uyemura, Taisa Magnani Dinamarco

**Affiliations:** 1 Faculty of Philosophy, Sciences and Literature of Ribeirão Preto, Chemistry Department, University of São Paulo, Ribeirão Preto, São Paulo, Brazil; 2 Faculty of Pharmaceutical Science, Department of Clinical, Toxicological and Bromatological Analysis, University of São Paulo, Ribeirão Preto, São Paulo, Brazil; 3 Brazilian Bioethanol Science and Technology Laboratory, Campinas, São Paulo, Brazil; 4 Current address: Laboratory of Regulatory Systems Biology, Department of Biochemistry, Institute of Chemistry, University of São Paulo, São Paulo, Brazil

**Keywords:** *Aspergillus fumigatus*, Sugarcane bagasse, CAZymes, Lignocellulose breakdown, RNA-Seq, Secretome

## Abstract

**Background:**

Sugarcane bagasse has been proposed as a lignocellulosic residue for second-generation ethanol (2G) produced by breaking down biomass into fermentable sugars. The enzymatic cocktails for biomass degradation are mostly produced by fungi, but low cost and high efficiency can consolidate 2G technologies. *A. fumigatus* plays an important role in plant biomass degradation capabilities and recycling. To gain more insight into the divergence in gene expression during steam-exploded bagasse (SEB) breakdown, this study profiled the transcriptome of *A. fumigatus* by RNA sequencing to compare transcriptional profiles of *A. fumigatus* grown on media containing SEB or fructose as the sole carbon source. Secretome analysis was also performed using SDS-PAGE and LC-MS/MS.

**Results:**

The maximum activities of cellulases (0.032 U mL-1), endo-1,4-β--xylanase (10.82 U mL-1) and endo-1,3-β glucanases (0.77 U mL-1) showed that functional CAZymes (carbohydrate-active enzymes) were secreted in the SEB culture conditions. Correlations between transcriptome and secretome data identified several CAZymes in *A. fumigatus*. Particular attention was given to CAZymes related to lignocellulose degradation and sugar transporters. Genes encoding glycoside hydrolase classes commonly expressed during the breakdown of cellulose, such as GH-5, 6, 7, 43, 45, and hemicellulose, such as GH-2, 10, 11, 30, 43, were found to be highly expressed in SEB conditions. Lytic polysaccharide monooxygenases (LPMO) classified as auxiliary activity families AA9 (GH61), CE (1, 4, 8, 15, 16), PL (1, 3, 4, 20) and GT (1, 2, 4, 8, 20, 35, 48) were also differentially expressed in this condition. Similarly, the most important enzymes related to biomass degradation, including endoxylanases, xyloglucanases, β-xylosidases, LPMOs, α-arabinofuranosidases, cellobiohydrolases, endoglucanases and β-glucosidases, were also identified in the secretome.

**Conclusions:**

This is the first report of a transcriptome and secretome experiment of *Aspergillus fumigatus* in the degradation of pretreated sugarcane bagasse. The results suggest that this strain employs important strategies for this complex degradation process. It was possible to identify a set of genes and proteins that might be applied in several biotechnology fields. This knowledge can be exploited for the improvement of 2G ethanol production by the rational design of enzymatic cocktails.

**Electronic supplementary material:**

The online version of this article (10.1186/s12864-018-4627-8) contains supplementary material, which is available to authorized users.

## Background

The demand for energy has increased continuously worldwide, which has raised concerns about sustainability and has prompted the search and development of advanced renewable and sustainable sources of energy [1]. Bioethanol has been noted as an alternative fuel to tackle these issues [1–3]. In Brazil, ethanol production relies on the fermentation of sucrose from sugarcane to yield the so-called first-generation (1G) bioethanol [4, 5]. The current Brazilian production is estimated at 30 billion liters per year, but the growing appeal of this fuel has called for investments in the development of new technologies to produce ethanol [6]. Large amounts of sugarcane straw and bagasse are generated every year in Brazil, so this biomass could be used as a substrate to produce 2G bioethanol, which in a few years will compete with 1G ethanol costs [6–11].

Lignocellulose is the most abundant material in nature. It consists of three major polymers: cellulose, hemicellulose and lignin. Cellulose, the main polymeric component of plant biomass, usually contains regions that are highly crystalline. It is a linear polymeric chain of over 10,000 D-glucose residues linked by β-1,4-glycosidic bonds [12–14]. The degradation of lignocellulose into fermentable sugars require many types of enzymes, e.g., β-glucosidases, cellobiohydrolases, endoglucanases, β-xylosidases, endo-β-1,4-xylanases, and numerous other auxiliary enzymes [5, 12, 15]. Due its recalcitrant characteristic, lignocellulose is difficult to degrade, even when enzymes work synergistically [12, 16].

Filamentous fungi such as *Trichoderma reesei* and *Aspergillus niger* play an important role in the secretion of enzymes known as CAZymes (carbohydrate-active enzymes), which can act synergistically and are the main source of enzymatic cocktails. Several studies have been conducted to optimize the current enzymatic cocktails and to reduce costs involved in 2G ethanol production [4, 12, 17–19]. The *Aspergillus* genus comprises over 250 species and has received much attention due numerous species secreting hydrolytic enzymes of interest to lignocellulosic biorefineries. *A. fumigatus* is an opportunistic and pathogenic fungus, and depending on immunological status of host, can lead to a variety of allergic reactions. However is an important producer of lignocellulolytic enzymes that act synergistically to increase the efficiency of the secreted enzymes. In addition, this fungus secretes thermostable glycosyl hydrolases, such as β-glucosidases (EC 3.2.1.21), endoglucanases (EC 3.2.1.4), cellobiohydrolases (EC 3.2.1.9), xylosidases (EC 3.2.1.37) and endoxylanases (EC 3.2.1.32), which can withstand elevated temperatures [20–23].

Previously, specific cellulose-, hemicellulose-, pectin-, and lignin-degrading enzymes were identified as secreted by *A. fumigatus* in the presence of different carbon sources (Avicel, cellulose, rice straw, starch, xylan, corn and soybean) that can be used in the lignocellulosic bioenergy industry [22, 24–26]. To gain more insight into how efficiently *A. fumigatus* AF293 can depolymerize the sugarcane bagasse, a complex biomass important for Brazilian 2G ethanol production, and to identify genes and proteins responsible for these lignocellulosic breakdown reactions, we examined the transcriptional response by RNA-Seq and proteomic profile by mass spectrometry (LC-MS/MS) of *A. fumigatus* that was cultivated in the presence of steam-exploded sugarcane bagasse (SEB).

## Methods

### Strains, media, and growth conditions

*A. fumigatus* AF293, gently donated by the Prof. Dr. Sérgio Akira Uyemura (University of São Paulo, BR), was grown on YAG medium (2% (*w*/*v*) dextrose, 0.5% (*w/v*) yeast extract, 0.1% (*v*/v) trace elements and 1.8% (*w/v*) agar) at 37 °C for two days. Spores were harvested and inoculated to a final concentration of 1 × 10^8^ per 50 mL of YNB culture with 1% (*w/v*) fructose as the carbon source at 37 °C for 16 h (h) in a rotary shaker with agitation at 200 rpm. Afterward, the mycelia were transferred to 1% (*w/v*) SEB (47.5% cellulose; 9.0% hemicellulose and 34.3% lignin) or 1% (*w/v*) fructose as the carbon source for 3, 6, 12, 18, 24, 48 and 72 h. Fructose was used as a control in all experimental conditions [26]. Mycelia were harvested by filtration through Whatman grade 1 filters (GE Healthcare, Grandview Blvd. Waukesha, WI, USA), washed once with sterile cool water and kept at − 80 °C until RNA extraction. Supernatants were collected to measure enzymatic activity and determine the secretome. All the experiments described below were performed in three biological replicates.

### Enzymatic activity assays

Specific xylanase (endo-1,4-β-xylanase) and cellulose (endo-1,4-β-glucanase) activities were performed with Azo-Xylan (Birchwood) and Azo-CM-Cellulose (both from Megazyme International, Bray, Ireland) as substrates, respectively, according to the manufacturer’s protocols. The enzymatic activities are represented as U mL^− 1^. All the reactions were performed in triplicate. The software Mega-Calc™ (Megazyme International) was used to determine the enzymatic activities.

Enzymatic activities were also measured by the dinitrosalicylic acid (DNS) assay [27]. Cellulase activities were measured with β-glucan and low-viscosity carboxymethylcellulose (CMC) as substrates, and xylanase activities were measured with the xyloglucan. Briefly, 20 μL of the supernatant from the samples grown in presence of 1% SEB for 24, 48 and 72 h were mixed with 30 μL of sodium acetate buffer 100 mM (pH 5.5) and 50 μL of substrate at 0.5% (*w*/*v*) final concentration to achieve a final volume of 100 μL. The reactions were incubated at 40 °C for 5 min for β-glucan and for 10 min for xyloglucan and CMC substrates. The reaction was stopped by adding 100 μL of DNS. All the reactions were performed in triplicate. The calculation of enzyme activities was based on a corresponding standard containing glucose. One unit (U) of enzymatic activity was defined as the amount of enzyme needed to liberate 1 μmol of reducing sugars per minute.

### RNA isolation and cDNA synthesis

Fungal biomass was harvested at different times from SEB or fructose culture conditions, and mycelia were ground in liquid nitrogen using a mortar and pestle. Total RNA was purified by using the “Direct-zol™ RNA MiniPrep” kit according to the manufacturer’s instructions (Zymo Research, Irvine, CA, EUA) using the on-column DNAse treatment. RNA integrity was confirmed with a bioanalyzer by using the “Agilent RNA 6000 Nano” kit (Agilent Technologies, Santa Clara, CA, EUA) and the “Plant Total RNA Nano” protocol. RNA was quantified on a Qubit® 2.0 fluorimeter (Thermo Fisher Scientific, Waltham, MS, EUA) with the Qubit® RNA BR Assay kit (Thermo Fisher Scientific, Waltham, MS, EUA). cDNA was synthesized from 1 μg of mRNA using SuperScript® II Reverse Transcriptase (Invitrogen, Carlsbad, CA, EUA).

### Library preparation and RNA sequencing

RNA sequencing libraries were prepared using the “TruSeq Stranded mRNA HT Sample Prep” kit (Illumina, San Diego, CA, EUA), mRNA enrichment was performed using magnetic beads coupled with oligo (dT). Sequencing was carried out in the HiSeq 2500 system (Illumina, San Diego, CA, EUA) at the NGS facility located at the Brazilian Bioethanol Science and Technology Laboratory (CTBE), Campinas, SP, Brazil.

### Bioinformatic analysis of RNA-Seq data

FastQC [28] was used to check the quality of the sequencing reads visually. Removal of the remaining adapter sequences and quality trimming with a sliding window of size 4, minimum quality of 20, and length filtering (to keep reads with a length of at least 60 bp) were performed with Trimmomatic v0.32, [29]. Clean reads were screened against a database of ribosomal RNA with the aid of SortMeRNA [30]. High-quality reads were mapped in a strand-specific manner by using TopHat2 [31] against the genome sequence of *A. fumigatus* Af293 obtained from ASPGD [32, 33]. The number of exon-exon junctions at different levels of read subsampling was employed to confirm sequencing saturation with RSeQC [34]. Mapping of the reads to the features of the exons were summarized at the gene level by using the function featureCounts from the Rsubread v1.12.6 package [35] in R v3.0.2 [36] and the annotation file in GFF3 format from ASPGD. Differential gene expression was analyzed with edgeR [37] in R [36]. Briefly, genes with at least one CPM (counts per million) in at least three samples were kept for analysis, which was equivalent to removing genes with low and noisy expression. The expression values were normalized by the trimmed mean of M-values (TMM) method to account for differences in the composition of RNA [38]. After the dispersion was estimated and the biological coefficient of variation was computed, the differentially expressed genes were called by fitting a negative binomial model with generalized linear models (GLS) that included factors for the TMM and the dispersion estimates [37]. A likelihood ratio test was performed to provide a *p*-value for differential expression. The *p*-values were adjusted for multiple testing by the method of Benjamini-Hochberg, to control the false discovery rate (FDR) [39]. The full R script used for the analysis and the raw count matrix are available in Additional file 1: Figure S1 and Additional file 2: Table S1, respectively. Genes with FDR values lower than 0.05 and log2-fold changes greater than 1.0 or lower than − 1.0, i.e., a difference of twice the expression level in either direction, were considered differentially expressed.

### Supernatant analysis by SDS-PAGE

Supernatants (50 mL) from SEB or fructose culture conditions were lyophilized until completely dry and re-suspended in 2 mL of buffer (Tris-HCl 50 mM, pH 6.8; 1 mM DTT; and 1 mM protease inhibitor), and 15 μL was separated by 10% SDS-PAGE (110 V, 90 min). The proteins were visualized by staining with 0.1% Coomassie Brilliant Blue R250 (*w*/*v*), which was followed by destaining with 45% methanol and 10% acetic acid solution (*v*/v). The protein concentration was determined by Bradford’s Assay (Bio-Rad Protein Assay Hercules, CA, EUA) [40]. Prior to mass spectrometry, all the bands from the SDS-PAGE gels were manually excised, reduced, alkylated, digested with trypsin, and purified (Promega, Madison, WI, EUA - V5111) according to a previously described method [41].

### Identification of proteins by coupled system of the LC-MS/MS type

Peptides were sequenced on a Synapt G2 HDMS (Waters, Milfords, MS, EUA) mass spectrometer coupled to a UPLC NanoAcquity system with 1D technology (Waters, Milfords, MS, EUA) and captured by a C18 Symmetry column (5 μm, 180 μm × 20 mm) (Waters, Milfords, MS, EUA). The peptides were separated by using a 2–90% acetonitrile gradient in 0.1% formic acid and an HSS T3 analytical column (1.8 μm, 75 μm × 100 mm) (Waters, Milfords, MS, EUA) with a flow of 300 μL min^− 1^ for 120 min. The data were acquired on a Waters Synapt G2S Q-TOF mass spectrometer equipped with a NanoLockSpray (Waters, Milfords, MS, EUA). The experiments were performed in the HDMSE mode (data-independent analysis). The mass spectra were processed with the ProteinLynxGlobalServer (PLGS) software version 3.1. The proteins were identified by comparison to the *Aspergillus* UNIPROT database (207,966 proteins) [42]. The defined parameters were automatic tolerance for precursors and ion products, minimum of three corresponding ion fragments per peptide, minimum of seven corresponding ion fragments per protein, trypsin missed cleavage, carbamidomethylation as a fixed modification, oxidation of methionine as a variable modification, and 4% FDR peptide.

### Protein analysis

Protein sequences were analyzed with the BLAST (basic local alignment search tool) software (http:ncbi.nlm.nih.gov/Blast.cgi). The subcellular localization of proteins was predicted by YLoc (interpretable subcellular localization prediction) (abi.inf.uni-tuebingen.de/Services/YLoc/webloc.cgi) [43], and the presence of signals due to peptides of the secreted proteins was predicted by SignalP v.4.0 (http://www.cbs.dtu.dk/services?SignalP/) [44]. Additionally, Secretome Pv2.0 (http://www.cbs.dtu.dk/services/SecretomeP/) was used to define the proteins that were secreted by the non-classic pathway [45].

For CAZy enzyme identification, the proteins in the secretome were screened with a library of hidden Markov models by using HMMER3 [46] of carbohydrate-active enzymes obtained from dbCAN [47]. Hits were considered positive on the basis of the dbCAN recommendations.

### qRT-PCR analysis

After RNA-Seq analysis, 4 DEGs (Differentially Expressed Genes) were selected, including sugar transporters and CAZymes, for qRT-PCR analysis. RNA was extracted and purified as previously described. cDNA was synthesized from 5 μg of RNA using SuperScript® II Reverse Transcriptase (Invitrogen, Carlsbad, CA, USA). Quantitative PCR (qPCR) analyses were performed according to Semighini et al. [48]. The abundance of the respective mRNAs was normalized using β-tubulin probes. The primers for the investigated genes are listed in Additional file 3: Table S2.

### Functional enrichment

Genes identified as differentially expressed were analyzed by FunCat functional enrichment [49]. The CAZy proteins from the secretome were classified according to the GO-Slim classifications from the AspGD based on the ontology “Molecular function” (GO Categorization Slim Mapper) [50].

### Venn diagrams

The area-proportional Venn diagrams were drawn based on images generated with free online software [51].

## Results

### Enzymatic analysis

**Fig. 1:**
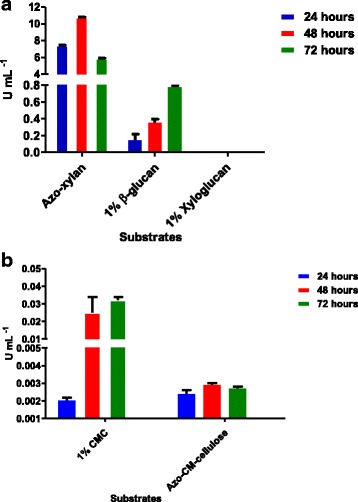
Enzyme activity. Enzymatic activities (U mL^−1^) of supernatants from *A. fumigatus* culture against different substrates after 24, 48 and 72 h growth on sugarcane bagasse. Each bar represents the mean and the standard deviation of values from three independent experiments. **a** Azo-xylan, β-glucan and xyloglucan; **b** 1% CMC and Azo-CMC

To evaluate the activity of enzymes produced by *A. fumigatus* in the presence of sugarcane bagasse, we performed enzymatic assays using xyloglucan, β-glucan, and CMC as substrates. Specific endo-1,4-β-xylanase and endo-1,4- β-glucanase activities, were also investigated using Azo-Xylan (Birchwood) and Azo-CM-Cellulose (both from Megazyme International, Bray, Ireland), respectively. The enzymes from supernatants derived from *A. fumigatus* cultures were capable of hydrolyzing cellulose (CMC) and hemicelluloses (β-glucan and xylan), but no activities were detected in xyloglucans (Fig. 1a-b).

We observed that the activities depended on duration of growth, with maximum activities detected within three days, except for endo-1,4-β-D-xylanase, with peak activity after two days culture and no activities detected prior to 24 h of incubation. Maximum activities of cellulases (0.032 U mL^− 1^), endo-1,4-β--xylanase (10.82 U mL^− 1^) and endo-1,3-β glucanases (0.77 U mL^− 1^) were detected for *A. fumigatus* while in *A. niger* (0.002 U mL^− 1^; 2.3 U mL^− 1^ and 0.4 U mL^− 1^, respectively) and *T. reesei* RUT-C30 (0.0039 U mL^− 1^, 0.4 U mL^− 1^ and 0.1 U mL^− 1^, respectively) are described on the same biomass [4]. These results indicate that *A. fumigatus* is an excellent producer of an arsenal of hydrolytic enzymes, with activities superior to the hypercellulolytic strain *T reesei* RUT30-C.

To gain more insight into the hydrolytic enzymes of *A. fumigatus* specific for sugarcane bagasse breakdown, we selected the cultivation of 24 h (when we detected enzyme activity) because we were interested in the initial process of SEB breakdown. In this time we determinate the transcriptome and the secretome responses of this strain.

### Analysis of the transcriptome of *A. fumigatus* under the influence of sugarcane bagasse as the substrate

To identify potential new genes involved in SEB breakdown, we analyzed the transcriptome by RNA-Seq after 24 h of cultivation. After RNA sequencing, each sample generated approximately 14 to 16 million paired-end reads. RNAseq data were analysed by comparing the mycelium grown on SEB and that grown on fructose. We observed 2227 genes differentially expressed (FDR < 0.05, |log2FC| > 1) in SEB where 1181 were upregulated, while 1045 were upregulated in fructose conditions (downregulated in SEB) (Additional file 4: Table S3). Gene ontology (GO) and the functional catalogue (FunCat) classified the differentially expressed genes functionally in 18 different enriched categories [40]. Two significant categories among upregulated genes were Metabolic Processes (GO:0008152) and Protein Synthesis (GO:0006412) and in downregulated genes Metabolic Processes (GO:0008152) and Energy (GO:0006112) (Additional file 5: Table S4). Genes related to the regulation of C-compound and carbohydrate metabolic processes represent the two GO terms commonly enriched for both up- and downregulated genes in the SEB condition, including genes encoding carbohydrate-active enzymes (CAZymes) and transporters. Given the importance of CAZymes for the degradation of biomass, we directed our efforts toward a better understanding of the transcriptional profile of these enzymes and sugar transporters.

**Fig. 2:**
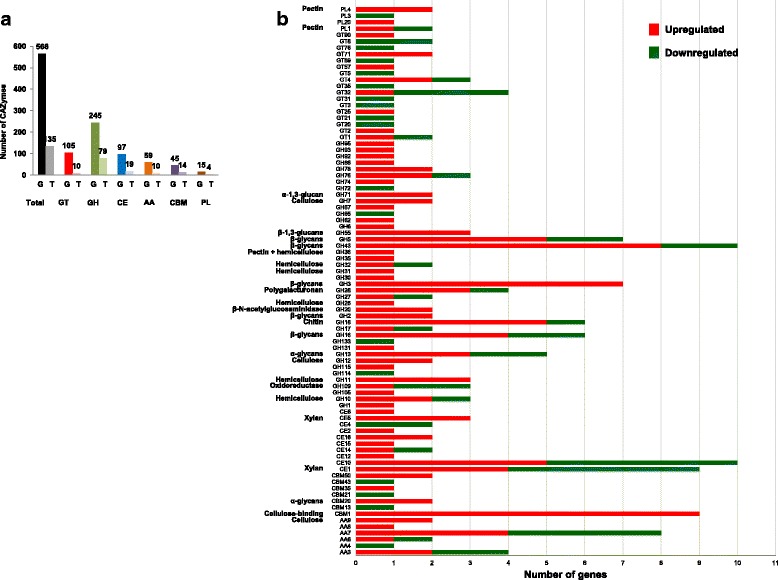
Differentially expressed CAZymes of *A. fumigatus* identified in RNA-Seq data. The total number of CAZymes and their respective families found in the genome (G) and upregulated in the transcriptome (T) (**a**). The classification of CAZymes families of up- and downregulated genes (**b**). AA, auxiliary activities; CBM, carbohydrate binding module; CE, carbohydrate esterase; GT, glycosyltransferases; PL, polysaccharide lyase; GH, glycoside hydrolases. Known substrates or activities of some CAZyme families are given

We found 197 differentially expressed CAZyme genes classified based on the CAZy database (http://www.cazy.org) [52]. Concerning the 566 CAZyme genes predicted in the *A. fumigatus* AF293 genome categorized into the different classes (247 GHs, 105 GTs, 96 CEs, 59 AAs, 15 PLs, and 44 CBMs) (Additional file 6: Table S5), we concluded that 35% of CAZyme genes were differentially expressed in our data, which highlights the potential that a wide spectrum of hydrolytic enzymes were produced. However, glycosyl transferases appeared only in a very small percentage (~ 1%), suggesting a secondary role in polysaccharide degradation (Fig. 2a).

Among the 197 CAZymes differentially expressed, 135 genes were upregulated in SEB and 62 were downregulated in SEB (upregulated in fructose), classified into 67 and 41 families, respectively (Additional file 4: Table S3). The classes of upregulated genes were 40% glycoside hydrolases (GH), 10% carbohydrate esterases (CE), 7% carbohydrate-binding modules (CBM), 5% glycosyltransferases (GT), 5% auxiliary activities (AA) and 2% polysaccharide lyases (PL), while downregulated genes represented 11% GHs, 6% CEs, 1% CBMs, 8% GTs, 4% AAs, and 1% PLs.

For plant biomass degradation, many enzymes working synergistically are required for efficiency hydrolysis. For cellulose degradation, endoglucanases (EGs) catalyze the hydrolysis at random positions in less crystalline regions; cellobiohydrolases (CBHs) act on the reducing and non-reducing ends of the chains, releasing cellobiose, which is cleaved into glucose by β-glucosidases [53]. We observed a synergistic upregulation of endoglucanases (GH5, GH12, GH16 and CBM1), cellobiohydrolases (GH6 and GH7) and β-glycosidases (GH1 and GH3). In addition to the cellulose degradation enzymes, 32 genes involved in xylan hydrolysis were also upregulated, e.g., endoxylanases (GH10, GH11 and CBM1), xylosidases (GH3 and GH43) and acetylxylan esterases (CBM1, CE2, CE16) (Fig. 2b).

In addition, numerous other plant cell wall polysaccharide-degrading enzymes were also upregulated as described in Additional file 4: Table S3. Among the DEGs, GH11 endo-1,4-beta xylanase (log_2_FC = 10.39) appeared highly expressed, as well as CBM1 endoglucanase (log_2_FC = 9.57), extracellular glycosyl hydrolase/cellulose CBM 1 (log_2_FC = 9.35) and AA9 endo-1,4-beta-glucanase (log_2_FC = 9.21 and 8.75) (Table 1). Genes encoding delignification enzymes, such as laccase (Afu2g17530), cellobiose dehydrogenase (Afu2g17620), catalase (Afu2g18030), putative FAD-dependent oxygenase (Afu6g12070), and oxidoreductase enzymes, were also upregulated. Similarly, pectate lyases – PL1 (Afu2g00760), amylases – GH13 (Afu2g03230), and carboxypeptidases (Afu3g07040 and Afu5g01200) were detected in this study (Additional file 4: Table S3).

- Taken together, endoglucanases, cellobiohydrolases and beta-glucosidases were significantly upregulated, suggesting the cellulose degradation potential of this strain, and the abundance of hemicellulases highlights, once again, the great potential of *A. fumigatus* in complex biomass deconstruction.

**Table 1:** Main CAZymes related to biomass deconstruction upregulated in *A. fumigatus* AF293 transcriptome

Gene ID	Gene Description	CAZy family	log_2_FC	Peptide Signal	Predicted substrate
Afu3g03870	endo-1,4-beta-glucanase	AA9	9.21	Y	cellulose
Afu4g07850	endoglucanase	AA9	8.75	Y	cellulose
Afu2g00920	extracellular glycosyl hydrolase/cellulase	CBM1	9.35	Y	arabinoxylan
Afu3g00420	acetyl xylan esterase (Axe1)	CBM1	4.13	Y	xylan
Afu6g01800	endoglucanase	CBM1	9.57	Y	cellulose
Afu6g03280	swollenin	CBM1	7.51	Y	cellulose
Afu6g11600	endoglucanase	CBM1	8.11	Y	cellulose
Afu6g13610	endo-1,4-beta-xylanase	CBM1	9.20	Y	xylan
Afu7g06740	endoglucanase	CBM1	8.34	N	cellulose
Afu8g06570	acetyl xylan esterase	CBM1	7.57	Y	xylan
Afu8g06830	endoglucanase	CBM1	4.40	Y	cellulose, β-1,4-glucan
Afu2g00690	glucan 1,4-alpha-glucosidase	CBM20	2.94	Y	starch
Afu4g10140	glucoamylase	CBM20	1.14	N	starch
Afu8g02510	glycosyl hydrolase family 43 protein	CBM35	1.61	Y	xylan, pectin
Afu2g14530	esterase	CE1	2.16	Y	xylan
Afu7g02380	ferulic acid esterase (FaeA)	CE1	1.72	Y	xylan
Afu2g00510	cellulose-binding GDSL lipase/acylhydrolase	CE16	7.28	Y	xylan, mannan
Afu2g00630	cellulose-binding GDSL lipase/acylhydrolase	CE16	3.97	Y	xylan, mannan
Afu2g09380	cutinase	CE5	7.10	Y	cutin
Afu2g14420	cutinase	CE5	3.71	Y	cutin
Afu4g03210	cutinase	CE5	6.74	Y	cutin
Afu1g14710	beta-glucosidase	GH1	3.42	N	cellulose
Afu3g15210	endo-1,4-beta-xylanase	GH10	8.59	Y	xylan
Afu4g09480	extracellular endo-1,4-beta-xylanase	GH10	8.82	Y	xylan
Afu3g00320	endo-1,4-beta-xylanase (XlnA)	GH11	10.39	Y	xylan
Afu3g00470	endo-1,4-beta-xylanase	GH11	8.64	Y	xylan
Afu6g12210	endo-1,4-beta-xylanase (XynG1)	GH11	6.91	Y	xylan
Afu7g06150	endoglucanase	GH12	8.58	Y	cellulose
Afu3g02090	beta-xylosidase	GH3	4.17	Y	xylan
Afu4g13770	glycosyl hydrolase	GH3	1.55	Y	cellulose
Afu5g07080	beta-glucosidase	GH3	2.03	Y	cellulose
Afu5g07190	beta-glucosidase	GH3	2.24	N	cellulose
Afu6g14490	beta-glucosidase	GH3	2.58	N	cellulose
Afu7g06140	beta-D-glucoside glucohydrolase	GH3	3.03	Y	cellulose
Afu8g02100	beta-glucosidase	GH3	2.13	Y	cellulose
Afu1g17320	endo-arabinanase	GH43	4.50	Y	pectin
Afu2g00930	xylosidase	GH43	7.97	N	xylan
Afu2g13190	xylosidase: arabinofuranosidase	GH43	1.99	N	xylan
Afu2g14750	endo-arabinase	GH43	1.92	Y	pectin
Afu3g01660	glycosyl hydrolase, family 43	GH43	2.24	Y	xylan, pectin
Afu6g00770	extracellular arabinanase	GH43	1.80	Y	xylan, pectin
Afu6g14550	xylosidase/arabinosidase	GH43	6.47	N	xylan, pectin
Afu8g04710	xylosidase	GH43	4.04	N	xylan
Afu5g01830	extracellular endoglucanase	GH5	2.00	Y	cellulose
Afu6g07480	endoglucanase	GH5	2.90	Y	cellulose
Afu7g01070	endo-1,4-beta-mannosidase	GH5	2.48	N	mannan
Afu7g05610	glucanase	GH5	5.07	N	β-1,6-glucan
Afu8g07030	endo-1,4-beta-mannosidase	GH5	1.82	Y	mannan, galactomannan, glucomannan
Afu3g01910	cellobiohydrolase	GH6	9.02	Y	cellulose
Afu2g12770	alpha-L-arabinofuranosidase	GH62	8.54	Y	arabinoxylan, arabinogalactan
Afu5g14380	Alpha-glucuronidase	GH67	2.58	Y	xylan
Afu6g07070	cellobiohydrolase celD	GH7	6.78	Y	cellulose
Afu6g11610	1,4-beta-D-glucan-cellobiohydrolyase	GH7	9.83	Y	cellulose
Afu8g01490	endoglucanase	GH74	7.89	Y	xyloglucan
Afu2g12830	UDP-glucosyl transferase family protein	GT1	1.81	N	UDP-glucosyl + acceptor
Afu8g02020	glycosyltransferase	GT2	3.09	N	–
Afu8g00650	LPS glycosyltransferase	GT25	2.11	N	UDP-glucose + lypopolysaccharide
Afu8g00640	glycosyl transferase	GT32	3.46	N	–
Afu1g06890	alpha-1,2-mannosyltransferase (Alg11)	GT4	1.01	N	GDP-mannose + Man3GlcNAc2-PP-dolichol or Man4GlcNAc2-PP-dolichol
Afu1g17030	glycosyl transferase	GT4	2.05	N	–
Afu3g07700	glucosyltransferase	GT57	1.31	N	dolichol-P-glucose + acceptor
Afu6g04450	alpha-1,2-mannosyltransferase (Mnn2)	GT71	1.46	N	GDP-mannose + heteroglycan
Afu6g14480	alpha-1,3-mannosyltransferase	GT71	1.22	N	GDP-mannose + heteroglycan
Afu2g00760	pectate lyase A	PL1	1.10	Y	pectin
Afu4g03780	rhamnogalacturonase B	PL4	1.76	Y	pectin
Afu8g00820	rhamnogalacturonase	PL4	3.09	Y	pectin

Modulated: all genes with log2FC > 1 and < −1 in presence of SEB

Up: genes with Log2FC > 1 in presence of SEB

Down: genes with Log2FC < − 1 in presence of SEB

**Fig. 3:**
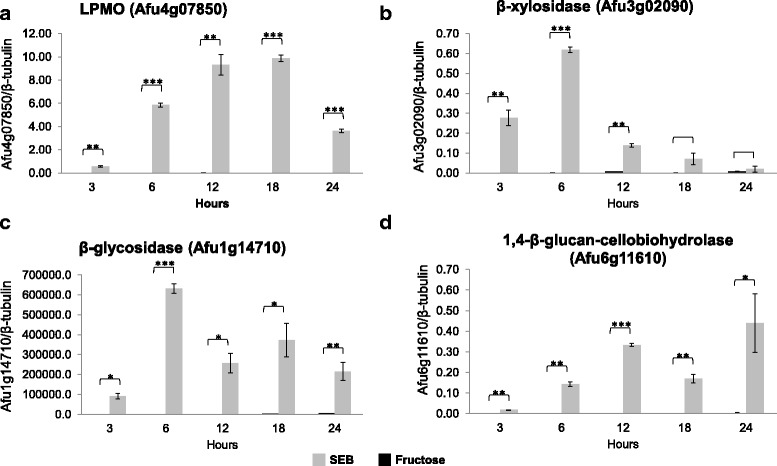
qRT-PCR. The expression levels of the Afu4g07850 (**a**), Afu3g02090 (**b**), Afu1g14710 (**c**), and Afu6g11610 (**d**) genes were determined after 3, 6, 12, 18, and 24 h of *A. fumigatus* growth in the presence of SEB 1% or fructose 1%. Each value represents the expression ratio relative to the expression of the β-tubulin gene. The data are the average of three replicates, and the bar indicates the standard deviation. Asterisks indicate significant differences (*, *P* < 0.05; **, *P* < 0.01; ***, *P* < 0.001) (Student’s t-test)

To validate RNAseq data and get additional information about the expression over time, we have performed qRT-PCR for 4 DEGs that encode enzymes essential to biomass degradation, Afu4g07850 (LPMO), Afu1g14710 (β-glucosidase), Afu6g11610 (1,4-β-D-glucan-cellobiohydrolase) and Afu3g02090 (β-xylosidase), during 3, 6, 12, 18 and 24 h of cultivation in SEB and fructose. The expression profiles of these genes behaved in different ways: Afu1g14710 and Afu3g02090 genes were strongly induced at the beginning (6 h) of the growth in SEB, and their expression decreased after 6 h, while Afu4g07850 had an increasing gene expression during the time course, and Afu6g11610 increased at 24 h (Fig. 3).

### Sugar transporters identified during RNA sequencing

**Fig. 4:**
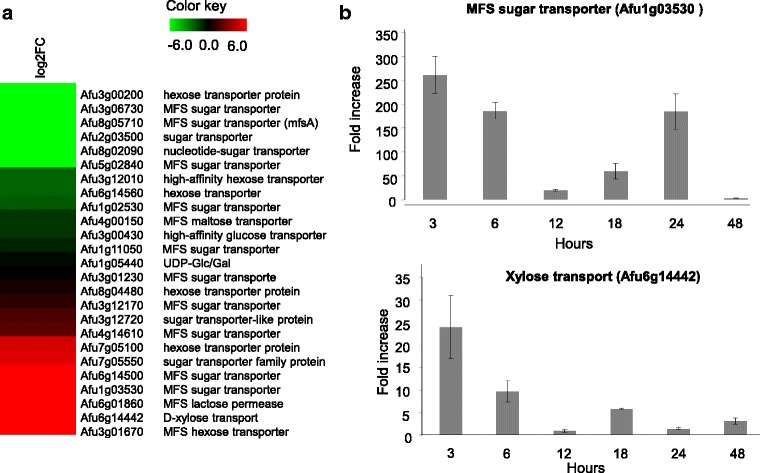
Sugar transporters differentially expressed in RNAseq. Heat map of up- and downregulated genes encoding sugar transporters (**a**). The color bar represents the log_2_FC values for each gene. Red color: upregulated genes; black color: unchanged genes; green color: downregulated genes. Expression profiles (qRT-PCR) of genes encoding sugar transporters (**b**) from *A. fumigatus* grown in the presence of 1% xylose. Each value represents a fold increase in the expression ratios compared to fungal growth in the presence of 1% fructose. Data are the average of three replicates, and the bar indicates the standard deviation

Approximately 106 genes encoding sugar transporters have been reported in the A*spergillus* genome, and only 88 genes were described as encoding sugar transporters in *A. fumigatus* strain Z5, which are distributed among the SP, FHS, SHS, and GPH families (the SP family includes 79 genes) [48]. Additionally, the genomes of filamentous fungi also encode large numbers of major facilitator superfamily (MFS) transporters. Among them, 25 transporters were differentially expressed on SEB, classified as encoding MFS hexose transporter, MFS and sugar transporter, UDP-Glc/Gal endoplasmic reticulum nucleotide sugar transporter, nucleotide sugar transporter, hexose transporter protein, high affinity glucose/hexose transporter, MFS glucose transporter, MFS lactose transporter, MFS maltose transporter, and xylose transporter (Fig. 4a).

Orthologous genes in *Aspergillus* and *non-Aspergillus* species were identified by sequence analysis according to *Aspergillus* Genome Database (AspGD; http://www.aspgd.org) [32]. Three orthologous genes encoding possible putative xylose transporters (Afu1g03530, Afu4g14610, and Afu6g14442) and five related to cellobiose transporters (Afu3g01670, Afu6g14500, Afu6g14560, Afu7g05100, and Afu8g04480) have been identified in *N. crassa*, *A. oryzae, A. niger*, and *A. nidulans* (Additional file 7: Table S6).

To analyze the potential xylose transporters, we selected Afu1g03530 (log_2_FC = 3.43) and Afu6g14442 (log_2_FC = 4.6), which are orthologous to the xtrD xylose transporter of *A. nidulans* (An0250) [54] and show high similarity to transporters in other fungi, with conserved regions among different species [54–69]. To characterize the expression profile, *A. fumigatus* was grown in 1% xylose and 1% fructose as a carbon source for the time course (3, 6, 12, 18, 24, and 48 h). Afu6g14442 gene expression was highly induced after 3 and 6 h of cultivation in 1% xylose with an increase in up to 25-fold. On the other hand, the expression of Afu1g03530 increased to 250-, 180-, 25-, 60-, 200-, and 5-fold after 3, 6, 12, 18, 24, and 48 h, respectively (Fig. 4b). These results lead us to speculate that both genes could encode potential xylose transporters, which can be further better characterized.

### Characterization of the secretome of *A. fumigatus* in the presence of sugarcane bagasse

**Fig. 5:**
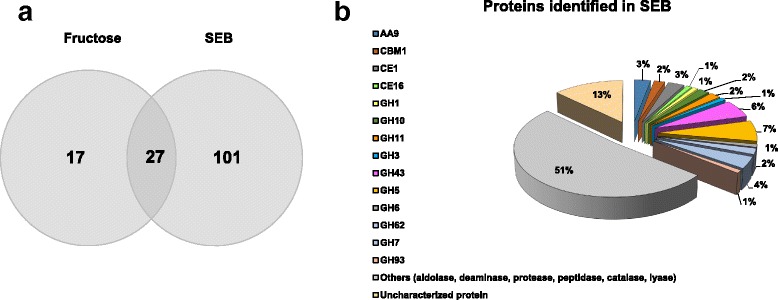
Proteins identified in the *A. fumigatus* secretome. Venn diagram of proteins found in the secretome of *A. fumigatus* grown on SEB or fructose (**a**). Percentage of CAZymes identified in the secretome classified according to their molecular function (**b**). CAZyme classification was from AspGD based on the facet of “Molecular function” [42]

Once the transcriptome was characterized, we analyzed the secreted protein profiles of *A. fumigatus* cultivated in the same condition by SDS-PAGE and LC-MS/MS. The total protein secreted by the fungi was approximately 300 μg mL^− 1^ in SEB versus 112 μg mL^− 1^ in the fungi grown on fructose (Additional file 8: Figure S2). In the SEB supernatant, we detected 128 secreted proteins, and only 44 were detected in the fructose supernatant (Additional file 9: Table S7), 27 of which are the same for both conditions (Fig. 5a).

As previously described, in the *A. fumigatus* genome, 566 CAZyme genes (461 proteins, excluding GTs) are predicted [25]; 271 of them are predicted to be secreted (Additional file 6: Table S5). The presence of a signal peptide in these proteins was inferred using SignalP 3.0 (http://www.cbs.dtu.dk/services/SignalP/) [44]. Approximately 78 proteins identified in SEB correspond to CAZymes in the classes GH (46), CE (8), AA (8), PL (2), and CBM (15), which is 18.61% of the total CAZymes encoded by the *A. fumigatus* genome. The remaining proteins are classified as proteins of unknown functions (17%), hydrolases/peptidases/proteases/binding proteins (14%), oxidoreductases (6%), transferases (2%), and lyases (2%) (Fig. 5b). We have also identified intracellular proteins, suggesting cell lysis or an unknown mechanism.

The most commonly identified cellulases and hemicellulases were GH5 and GH43, respectively. When compared to previous data concerning the secretome of *A. fumigatus,* in different types of biomass [22–24]*,* we detected CAZymes found exclusively in the SEB supernatant, such as GH10 (endo-1,4-β-xylanase), GH11 (Endo-1,4-β-xylanase (xyn11A), GH43 (β-glucosidase), GH43 (arabinase), GH47 (mannosidase), GH5 (endo-1,4-β-mannosidase) GH28 (xylogalacturonan), GH27 (α-galactosidase) GH62 (α-arabinofuranosidase) PL3 (pectate lyase) and CE1 (acetyl xylan esterase) (Table 2). Table S7 lists all these enzymes identified in both SEB and fructose conditions.

**Table 2:** CAZymes detected in *A. fumigatus* SEB secretome related to biomass breakdown

Uniprot Acession	Gene ID	Protein Name	CAZy Family	Score	SeqCover (%)	Peptide Signal	Substrate
Q6MYM8	Afu1g12560	endoglucanase	AA9	1158.51	13.67	Y	cellulose
Q4WP32	Afu4g07850	endoglucanase^a,c^	AA9	11,713.03	47.2	Y	cellulose
Q4X071	Afu2g14490	endoglucanase	AA9	1475.28	15.22	Y	cellulose
D4AHU7	Afu6g03280	swollenin	CBM1	7631.56	32.14	Y	cellulose
Q4WBW4	Afu8g06570	acetyl xylan esterase	CBM1	5408.98	9.16	Y	xylan
B0Y7U1	Afu6g09040	feruloyl esterase	CE1	250.94	11.22	Y	arabinoxylan, pectin
A4D9B6	faeC	feruloyl esterase C	CE1	3223.58	37.87	Y	xylan
Q4WIS4	Afu2g00820	extracellular GDSL-like lipase/acylhydrolase^a^	CE16	3089.24	22.45	Y	xylan, mannan
Q4WRY0	Afu1g14710	beta-glucosidase^a^	GH1	4556.9	26.5	N	cellulose
Q4WCM9	Afu6g01800	endoglucanase^a^	GH7/CBM1	6457.03	16.74	Y	cellulose
Q0H904	Afu4g09480	endo-1,4-beta-xylanase C (xlnC)^a^	GH10	34,440.44	85.23	Y	xylan
Q4WLG5	Afu6g13610	endo-1,4-beta-xylanase^a,c^	GH10/CBM1	7068.88	58.19	Y	xylan
V5R355	Afu3g00320	endo-1,4-beta-xylanase (XlnA)^a,c^	GH11	10,011.55	37.04	Y	xylan
B0Y8Q8	Afu6g12210	endo-1,4-beta-xylanase (XynG1)	GH11	1009.69	15.38	Y	xylan
Q4WQR8	Afu4g13770	glycosyl hydrolase	GH3	327.91	7.06	Y	cellulose
B0YDT3	Afu6g00770	extracellular arabinanase	GH43	4307.26	28.04	Y	xylan, pectin
Q4WIR3	Afu2g00930	xylosidase/glycosyl hydrolase^a,c^	GH43	2555.85	13.28	N	xylan
Q4WIU1	Afu2g00650	arabinosidase^a,c^	GH43	8662.27	27.8	N	pectin
Q4X046	Afu2g14750	endo-arabinase^b^	GH43	2990.38	34.88	Y	pectin
Q4WCE5	Afu8g04710	xylosidase^a^	GH43	2270.88	24.77	N	xylan
Q4WBJ5	Afu8g02510	glycosyl hydrolase family 43 protein^c^	GH43/CBM35	583.34	22.2	Y	xylan, pectin
Q4WD15	Afu6g03150	Uncharacterized protein, hydrolase activity^a^	GH5	2751.27	17.67	Y	unknown
Q4WW63	Afu5g14560	Cellulase family protein^b^	GH5	2371.35	20.15	Y	cellulose
Q4WGN1	Afu7g05610	glucanase^a^	GH5	1540.22	16.6	N	β-1,6-glucan
Q4WN62	Afu6g07480	endoglucanase	GH5	2409.74	28.1	Y	cellulose
B0Y9E7	Afu8g07030	endo-1,4-beta-mannosidase	GH5/CBM1	649.86	16.21	Y	mannan, galactomannan, glucomannan
F1DGF4	Afu6g11600	endoglucanase^a,c^	GH5/CBM1	8172.11	36.52	Y	cellulose
Q4WE56	Afu5g01830	extracellular endoglucanase^a^	GH5/CBMX2	6764.9	19.83	Y	cellulose
B0XWL3	Afu3g01910	cellobiohydrolase^a,c^	GH6/CBM1	6069.09	23.13	Y	cellulose
Q4X0P5	Afu2g12770	alpha-L-arabinofuranosidase^a^	GH62	9479.28	40.66	Y	arabinoxylan, arabinogalactan
Q4WIR4	Afu2g00920	extracellular glycosyl hydrolase/cellulase^a,c^	GH62/CBM1	3622.83	28.28	Y	arabinoxylan
B0Y793	Afu6g07070	cellobiohydrolase celD^a,c^	GH7	21,820.01	54.65	Y	cellulose
Q4WM08	Afu6g11610	1,4-beta-D-glucan-cellobiohydrolyase^a,b^	GH7/CBM1	24,706.69	35.53	Y	cellulose
T1YVP0	N/A	Glucanase^a^	GH7/CBM1	6572.88	24.78	Y	cellulose
Q4WLW1	Afu6g12120	BNR/Asp-box repeat domain protein	GH93	778.23	16.32	Y	pectin

^a^Proteins identified in this study, which have also been identified in a previous study [23]

^b^Proteins identified in this study, which have also been identified in a previous study [22]

^c^Proteins identified in this study, which have also been identified in a previous study [24]

We have also identified lignin-depolymerizing enzymes such as catalase-peroxidase, cellobiose dehydrogenase, catalase B, FAD-dependent oxidase, laccase, and Cu-Zn superoxide dismutase. Although the sugarcane bagasse employed here was treated by steam explosion, traces of lignin might have remained in the substrate, which justifies the secretion of these enzymes by the fungus.

We can conclude that the most important CAZymes (GH3, GH5, GH6, GH7, GH10, GH11, GH43, GH62, GH93, CE1, CE16 and AA9 (LPMO)) were secreted and play important roles in biomass degradation. For the first time, new proteins such as GH16 (endo-1,4-beta-glucanase), GH5 (endoglucanase), LPMO (AA9), swollenin and GH3 (β-glucosidase) were identified in the *Aspergillus fumigatus* secretome, probably because we used sugarcane bagasse as the source of carbon, and these enzymes can be specific to this complex biomass.

### Integration of secretomics and transcriptomics

**Fig. 6:**
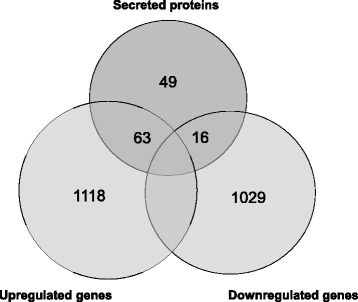
Intersection of differentially expressed genes and secreted proteins. Venn diagram of transcriptome (upregulated genes and downregulated genes) and proteins secreted by *A. fumigatus* when grown on SEB

We observed weak correlations between transcriptome and secretome datasets, mainly because we chose the same time (24 h) to isolate mRNA and proteins. Considering that *Aspergillus* needs at least a few hours to translate mRNA to protein and to secrete it, these data provide an idea about which proteins are transcribed earlier or produced constitutively. Among the 1181 upregulated genes in transcriptome, 63 encoded proteins were detected in the secretome. As the same way, 16 of secreted proteins were identified as downregulated in RNAseq data (Fig. 6). In addition, the weak correlation observed could be the result of the influence of some factors that alter transcription and translation mechanisms [22, 23, 54].

The both data revealed a significant upregulation of secreted CAZymes, which is important for the observation of specific alterations triggered by different conditions. Among the 135 upregulated CAZyme genes, 48 encoded proteins were detected in the secretome, mainly cellulolytic and hemicellulolytic enzymes (Table 3). Four enzymes, β-1,3-endoglucanase EglC (Afu3g00270), FAD-dependent oxygenase (Afu3g00840), FAD/FMN-containing isoamyl alcohol oxidase MreA (Afu6g03620) and oligopeptidase family protein (Afu8g04730), were downregulated when the strain was cultivated in SEB but were detected in the secretome.

**Table 3:** CAZymes identified in both transcriptome and secretome of *A. fumigatus* grown on SEB

Gene ID	Gene Description	CAZy family	log_2_FC
Afu3g00320	endo-1,4-beta-xylanase (XlnA)	GH11	10.39
Afu6g11610	1,4-beta-D-glucan-cellobiohydrolyase	GH7	9.83
Afu6g01800	endoglucanase	CBM1	9.58
Afu2g00920	extracellular glycosyl hydrolase/cellulase	CBM1	9.35
Afu6g13610	endo-1,4-beta-xylanase	CBM1	9.21
Afu6g12120	BNR/Asp-box repeat domain protein	GH93	9.07
Afu3g01910	Putative cellobiohydrolase	GH6	9.02
Afu4g09480	extracellular endo-1,4-beta-xylanase	GH10	8.82
Afu4g07850	endoglucanase	AA9	8.75
Afu2g12770	alpha-L-arabinofuranosidase	GH62	8.54
Afu6g11600	endoglucanase	CBM1	8.11
Afu2g00930	xylosidase/glycosyl hydrolase	GH43	7.97
Afu3g03080	Endo-1,3(4)-beta-glucanase, putative	GH16	7.70
Afu8g06570	acetyl xylan esterase	CBM1	7.57
Afu6g03280	swollenin	CBM1	7.51
Afu8g06890	Probable endo-xylogalacturonan hydrolase A (xghA)	GH28	6.99
Afu6g12210	endo-1,4-beta-xylanase (XynG1)	GH11	6.91
Afu6g07070	cellobiohydrolase celD	GH7	6.78
Afu5g01190	Uncharacterized protein, alpha-L-fucosidase activity	GH65	6.49
Afu2g17620	Cellobiose dehydrogenase	AA8	6.45
Afu2g15420	Uncharacterized protein	GH131	6.41
Afu6g14540	Endo-1,3(4)-beta-glucanase, putative	GH16	6.37
Afu8g01410	Endochitinase B1 (chiB1)	GH18	5.79
Afu2g03980	Alpha-1,3-glucanase/mutanase, putative	GH71	5.38
Afu3g14510	Rhamnogalacturonan acetylesterase (RgaE)	CE12	5.19
Afu7g05610	glucanase	GH5	5.07
Afu7g05140	Class III chitinase, putative	GH18/CBM19	4.86
Afu3g07160	Class V chitinase, putative	GH18	4.25
Afu3g00420	acetyl xylan esterase (Axe1)	CBM1	4.13
Afu8g04710	xylosidase	GH43	4.04
Afu6g12070	FAD binding domain protein	AA7	3.48
Afu1g14710	beta-glucosidase	GH1	3.42
Afu6g10130	N, O-diacetyl muramidase, putative	GH25	3.23
Afu1g14450	Exo-1,3-beta-D-glucanase, putative (exgO)	GH55	3.19
Afu2g00690	glucan 1,4-alpha-glucosidase	CBM20	2.94
Afu5g10930	Uncharacterized protein	PL20	2.91
Afu6g07480	endoglucanase	GH5	2.9
Afu5g10520	Alpha-1,2-mannosidase family protein	GH92	2.86
Afu8g07120	Endo-1,6-beta-D-glucanase neg1	GH30	2.76
Afu2g14520	Hydrolase, putative	GH2	2.67
Afu1g05290	Endo-1,3(4)-beta-glucanase, putative	GH16	2.38
Afu2g14530	esterase D	CE1	2.16
Afu5g01830	extracellular endoglucanase	GH5	2
Afu2g14750	endo-arabinase	GH43	1.92
Afu8g07030	endo-1,4-beta-mannosidase	GH5	1.82
Afu8g02510	glycosyl hydrolase family 43 protein	CBM35	1.61
Afu4g13770	glycosyl hydrolase	GH3	1.55
Afu6g02560	Alpha-galactosidase	GH27	1.36

The variation in expression during fungal growth in the presence of SEB was observed for some genes in the qRT-PCR data (Fig. 3). We observed that the gene expression depends on the duration of incubation, which explains the low correlation between the data of the secretome and transcriptome and supports the hypothesis that the approach used in this study is able to provide information about the modulation of gene expression of *A. fumigatus* in the presence of sugarcane bagasse. Genes encoding enzymes that are upregulated in the transcriptome (the highest log_2_FC) and are present in the proteome, as well, are described in Table 3. The main enzymes produced by the fungus are endo-β1,4-xylanase, cellobiohydrolase B, endoglucanase and arabinofuranosidase, which leads us to speculate that *A. fumigatus* spent its energy on the transcription and production of cellulolytic and xylanolytic compounds, which were probably being secreted for biomass degradation and the release of small sugars.

## Discussion

In Brazil, sugarcane bagasse is an important agro-industrial residue. It is composed of cellulose (25–45%), hemicellulose (28–32%), lignin (15–25%) and a small percentage of other compounds [70, 71]. Despite this recalcitrant composition, efficient hydrolysis mechanisms allow the release of fermentable sugars from sugarcane bagasse and their later use to produce cellulosic ethanol [18, 72–74]. Transcriptomic and/or proteomic studies on filamentous fungi have been employed to understand and to improve enzymatic cocktails to deconstruct plant biomass. Such studies have revealed a huge repertoire of cellulases and hemicellulases [4, 18, 19, 75–77]. *N. crassa, A. niger, T. reesei and A. nidulans* are excellent enzyme producers for industrial applications, and several studies have focused on these fungi [78–82]. Understanding the molecular mechanisms through which fungi degrade plant biomass can improve the SEB saccharification step, which is important for Brazilian 2G ethanol production [4, 18, 19, 83, 84].

To gain more insight into new enzymes and to identify new genes specific to sugarcane biomass hydrolysis, we have chosen to investigate *A. fumigatus* because it is widely distributed in the environment, it can degrade plant biomass, and it is an excellent enzyme producer [21]. To the best of our knowledge, this is the first study on transcriptional response and secretome of *A. fumigatus* grown on sugarcane steam-exploded bagasse.

There are few omic studies on biomass hydrolysis by *A. fumigatus* [22–25], and only one study has analyzed the transcriptome of *A. fumigatus* when grown on polysaccharide substrates. Miao et al. [25] conducted a transcriptional study on the induction of CAZymes by this fungus grown on cellulose, oat spelt xylan, rice straw and sucrose. The authors showed that important genes are differentially expressed in each carbon source.

Here, we found few discrepancies in the number of induced genes when we used SEB as carbon source (Table 4). The main CAZyme families (GH1, GH3, GH5, GH6, GH7, GH10, GH11, GH12, GH43, GH62, GH67, GH74, AA9, CE3, CE5 and CE16 [85]) were also induced. However, important genes including GH45, GH51, GH54, GH93, GH115, and CE1 were downregulated in SEB. Furthermore, two important genes were exclusively induced in SEB: PL4 (Afu4g03780 and Afu8g00820), which plays a role in pectin breakdown, and CBM35 (Afu8g02510), which is known to bind primarily to xylan and mannans [86]. The distinct gene expression was most likely due to substrate composition and to cultivation time, which was 24 h for our analysis. Shorter cultivation times could point out new pattern to gene induction. In qRT-PCR data, we observed that 6 h is the beginning of gene expression, which might represent a standard mechanism in which *A. fumigatus* acts in contact with complex biomass sources and should contain more highly induced enzymes. These results also explain the percentage (35%) of differentially expressed CAZymes identified in the RNA-Seq data at 24 h.

**Table 4:** *Aspergillus fumigatus* transcriptome studies using different carbon sources

Strain	Technology	Carbon source	Time	Total number of Cazymes genes upregulated	Reference
*A. fumigatus* Z5	Illumina	cellulose, xylan, rice straw, oat spelt	16 h	47, xylan; 143, rice straw; 157, cellulose	[25]
*A. fumigatus* AF293	Illumina	sugarcane exploded bagasse	24 h	135, SEB	This work

Because glycosyltransferases (GTs) contribute to fungal cell remodeling, the percentage of upregulated genes was low (~ 1%). Likewise, Miao et al. [25] described that genes encoding GTs are downregulated in Z5 strain, which supports the idea that GTs do not directly participate in the hydrolysis of complex biomass.

In this sense, the biomass itself has to be investigated in order to understand sugarcane biomass hydrolysis as well as possible. A similar work performed by Borin et al., 2017 [18] described a transcriptional response of *A. niger* and *T. reesei* grown in SEB for different periods. They found 190 upregulated CAZymes from 62 different families in *A. niger*, and 105 genes of 51 CAZyme families in *T. reesei*, whereas we detected 135 upregulated CAZymes in the *A. fumigatus* transcriptome herein. The number of genes induced by each microorganism was different and depended on time. A higher number of DEGs in *A. niger* and *T. reesei* was observed in 24 h of culture, and so we then compared our data at this duration, and again small differences in upregulated CAZymes were observed.

After biomass hydrolysis, which breaks down cellulose and hemicellulose into mono- or disaccharides, the released sugars need to be transported into the cells through a large number of sugar transporters, most of which have not been characterized yet [20, 21, 87]. One of the main challenges concerning biofuel production from lignocellulosic biomass is the inability of organisms to grow on, to transport, and to ferment sugars other than glucose (e.g., xylose and cellobiose). Gaining a better insight into potential xylose and/or cellobiose transporters seems to be a good approach to overcome this challenge [87]. These transporters represent an important industrial tool that can be applied to different industrial processes [88–90]. Additionally, the genomes of filamentous fungi also encode large numbers of major facilitator superfamily (MFS) transporters. For example, the *T. reesei* and *A. nidulans* genomes have been predicted to encode 164 and 357 proteins belonging to MFS, respectively, although the exact number of proteins involved in sugar transport remains unknown [91–93].

We were also interested in new sugar transporters, such as the xylose transporter. Until now, no sugar transporter for *A. fumigatus* related to biomass breakdown has been described. We verified that the 25 DEG homolog transporters had particular expression profiles, upregulated or downregulated, suggesting that SEB hydrolysis released enough glucose, xylose or cellobiose to regulate sugar transporter gene expression. Another interesting finding was that two sugar transporters (Afu6g14442 and Afu1g03530) were highly induced in SEB (25.5 times and 10 times, respectively) and in the presence of 1% xylose, which revealed that these sugar transporters could be specific xylose transporters in *Aspergillus fumigatus.* Overexpression of xylose transporters in *S. cerevisiae* is a fast way to use xylose and may improve ethanol productivity [94].

In addition to the transcriptome, we evaluated the proteins secreted by *A. fumigatus* by SDS-PAGE and LC-MS/MS. Similarly, three studies compared the secretome of *A. fumigatus* on complex substrates (Table 2) [22–24]. Liu et al., [24] identified 152 proteins on rice straw and 125 different proteins on Avicel. Adav et al., [23] quantified 73 proteins belonging to cellulases, glycoside hydrolases and amylases. We detected some secreted proteins that were also identified when *A. fumigatus* was grown on corn, wheat, soybean, Avicel, rice straw, xylan, and starch as carbon source. However, for the first time, we verified important secreted CAZymes like swollenin (CBM1), two putative endoglucanases (LPMO) (AA9), acetyl xylan esterase (CBM1), two feruloyl esterases (CE1), endo-1,4-beta-xylanase (GH11), endo-1,3(4)-beta-glucanase (GH16), glycosyl hydrolase (GH3), endoglucanase (GH5), arabinanase (GH43), endo-1,4-beta-mannosidase (GH5/CBM1), and arabinogalactan endo-1,4-beta-galactosidase (GH53). In this way, we can conclude that we detected similar amounts of proteins with those previously described, which may be specific for SEB biomass.

These data showed that, although the transcriptome data did not reveal potential new enzyme targets for the deconstruction of sugarcane biomass deconstruction, the secretome analyses indicated key enzymes that may be essencial for this hydrolysis and which act synergistically for efficient deconstruction. We clearly observed the need for accessory enzymes secreted as LPMO and swollenin, which have never been described in other *A. fumigatus* secretome analyses [95], which allowed us to conclude that the other secreted CAZymes together with AA9 identified in *A. fumigatus* form a potential arsenal of hydrolytic enzymes.

LPMOs have recently been implicated in lignocellulosic biomass degradation. Although these enzymes were first classified into the GH61 and CBM33 families, they are currently classified into the AA9 and AA10 families. These enzymes cleave the lignocellulosic biomass glycosidic bonds through an oxidative mechanism that provides new ends for the recognition of cellulases and for action on cellulose [95, 96]. In addition, AA9s have been identified in *A. niger*, *M. thermophile*, *T. asperellum*, *and T. reesei* secretome growth in sugarcane bagasse, which allows us to conclude that they play an important role in lignocellulosic biomass breakdown [4, 95–99].

Many studies have focused on LPMO enzymes, and some works have even characterized them, but no investigations into LPMO enzymes from *A. fumigatus* are available so far [96]. Hence, the role of most of these enzymes remains unclear, and AA expression during *A. fumigatus* growth on bagasse suggests that they play an important part in biomass degradation.

Together, the transcriptome and secretome have shown several enzymes that *A. fumigatus* uses to hydrolyze SEB and which most likely act synergistically to depolymerize cellulose and hemicellulose. In most *Aspergillus* species*,* distinct genes encode the same class of enzymes (isoenzymes) [77], as observed by the data regarding the *A. fumigatus* secretome and transcriptome. Our results suggest that complete hydrolysis of this lignocellulose biomass to simple sugars, such as glucose, xylose, and arabinose, requires the combined actions of several enzymes that have different substrate specificities and act synergistically. The great potential of this species is evident, and its enzymes can contribute to optimization of enzymatic cocktails for use in 2G bioethanol production.

## Conclusion

Through these findings, it is suggested that different biomasses require a set of enzymes due its complexity and *A. fumigatus* Af293 is an excellent CAZymes producer for sugarcane biomass breakdown. The analysis of proteome and transcriptome revealed a set of CAZymes highly expressed and secreted, such as cellulases, hemicellulases, delignification and auxiliary enzymes necessary to SEB breakdown. In addition, from CAZymes proteins, LPMOs, which could contribute to better degradation of cellulose, were also detected in *A. fumigatus* secretome. Cellobiohydrolases, endoglucanases and LPMOs can act synergistically in cellulose depolymerization and LPMOs can be included in the most advanced enzymatic cocktails. Altogether, the data show that despite the pathogenicity of *A. fumigatus*, it can produce a wide variety of enzymes, which can be expressed in a nonpathogenic microorganism and may contribute to the optimization of currently marketed enzymatic cocktails for the viable production of 2G bioethanol.

## Additional files

Additional file 1: **Figure S1.** R script of RNA sequencing analysis. (PDF 2406 kb)

Additional file 2: **Table S1.** Raw Count matrix of genes found in RNA-seq. (XLSX 585 kb)

Additional file 3: **Table S2.** Sequence of primers from genes upregulated in RNA-seqdata selected for qRT-PCR. (XLSX 11 kb)

Additional file 4: **Table S3.** Up and downregulated genes in SEB. (XLSX 314 kb)

Additional file 5: **Table S4.** Functional enrichment analysis with FunCat of up- and downregulated genes in *Aspergillus fumigatus* grown in SEB. (XLSX 12 kb)

Additional file 6: **Table S5.** CAZymes families in *A. fumigatus* genome and predicted peptide signal. (XLSX 32 kb)

Additional file 7: **Table S6.** *A. fumigatus* sugar transporters modulated in RNA-seq data. (XLSX 13 kb)

Additional file 8: **Figure S2.** Proteins from *A. fumigatus* secretome separated by SDS-PAGE. (PPTX 283 kb)

Additional file 9: **Table S7.** Proteins detected in the secretome of *Aspergillus fumigatus* grown in SEB by coupled system type LC-MS/MS. (XLSX 37 kb)

## Abbreviations

ASPGD: *Aspergillus* genome database
bp: Base pair
CAZymes: Carbohydrate-active enzymes
cDNA: Complementary DNA
CMC: Carboxymetilcellulose
CPM: Counts per million
DEGs: Differentially expressed genes
DNA: Deoxyribonucleic acid
DNS: Dinitrosalicylic acid
DTT: Dithiothreitol;
FDR: False rate discovery
FPKM: Fragments per kilobase of exon per million mapped reads
HCl: Hydrocloric acid
kb: Kilobases
log_2_FC: Log of fold-change in base 2
LPMO: Lytic polysaccharides mono-oxygenase
M: Molar (mol L^− 1^)
m/z: Mass/charge relation
min: Minute
mM: Millimolar
mRNA: Messenger RNA
NGS: New generation sequencing
PCR: Polymerase chain reaction
pH: Hydrogenionic potential
qRT-PCR: Real-time quantitative PCR
RNA: Ribonucleic acid
RNASeq: RNA sequencing
rpm: Rotations per minute
SDS: Sodium dodecyl sulphate
SDS-PAGE: SDS-polyacrilamide gel electrophoresis
SEB: Steam-exploded bagasse
Tris: Tris- (hydroxymethyl) aminoethane
U: Units
V: Volts
*v*/v: Volume/volume ratio
*w*/*v*: Weight/volume ratio
μg: Micrograms
μM: Micromolar

## References

[1] Souza GM, Victoria RL, Joly CA, Verdade LM (2015). Bioenergy & Sustainability: bridging the gaps.

[2] Cheng JJ, Timilsina GR (2011). Status and barriers of advanced biofuel technologies: a review. Renew En.

[3] Moysés DN, Reis VC, de Almeida JR, de Moraes LM, Torres FA (2016). Xylose fermentation by *Saccharomyces cerevisiae*: challenges and prospects. J Mol Sci.

[4] Borin GP, Sanchez CC, de Souza AP, de Santana ES, de Souza AT, Leme AFP (2015). Comparative secretome analysis of *Trichoderma reesei* and *Aspergillus niger* during growth on sugarcane biomass. PLoS One.

[5] Novacana. http://www.novacana.com (2016) Accessed 20 Nov 2016.

[6] Conab. http://www.conab.gov.br (2017) Accessed 10 Nov 2017.

[7] Milanez AY, Nyko D, Valente MS, de Sousa LC, Bonomi A, de Jesus CDF (2015). De promessa a realidade: como o etanol celulósico pode revolucionar a indústria da cana-de-açúcar: uma avaliação do potencial competitivo e sugestões de política pública. BNDES Setorial, Rio de Janeiro.

[8] Batalha LAR, Han Q, Jameel H, Chang H, Colodette JL, Gomes FJB (2015). Production of fermentable sugars from sugarcane bagasse by enzymatic hydrolysis after autohydrolysis and mechanical refining. Bioresour Technol.

[9] Pereira SC, Maehara L, Machado CM, Farinas CS. 2G ethanol from the whole sugarcane lignocellulosic biomass. Biotechnol Biofuels. 2015;8:44–60. 10.1186/s13068-015-0224-0 PMC4359543 25774217

[10] Sindhu R, Binod P, Pandey A (2016). Biological pretreatment of lignocellulosic biomass - an overview. Bioresour Technol.

[11] Sambusiti C, Licari A, Solhy A, Aboulkas A, Cacciaguerra T, Barakat A (2015). One-pot dry chemo-mechanical deconstruction for bioethanol production from sugarcane bagasse. Bioresour Technol.

[12] Glass NL, Schmoll M, Cate JHD, Coradetti S (2013). Plant cell wall deconstruction by ascomycete fungi. Annu Rev Microbiol.

[13] Wang Y, Fan C, Hu H, Li Y, Sun D, Wang Y, Peng L (2016). Genetic modification of plant cell walls to enhance biomass yield and biofuel production in bioenergy crops. Biotechnol Adv.

[14] Sun Y, Cheng J (2002). Hydrolysis of lignocellulosic materials for ethanol production: a review. Bioresour Technol.

[15] Behera S, Arora R, Nandhagopal N, Kumar S (2014). Importance of chemical pretreatment for bioconversion of lignocellulosic biomass. Renew Sustain En Rev.

[16] Cragg SM, Beckham GT, Bruce NC, Bugg TD, Distel DL, Dupree P (2015). Lignocellulose degradation mechanisms across the tree of life. Curr Opin Chem Biol.

[17] Sharma RK, Arora DS (2015). Fungal degradation of lignocellulosic residues: an aspect of improved nutritive quality. Crit Rev Microbiol.

[18] Borin GP, Sanchez CC, Santana ES, Zanini GK, dos Santos RAC, Pontes AO (2017). Comparative transcriptome analysis reveals different strategies for degradation of steam-exploded sugarcane bagasse by *Aspergillus niger* and *Trichoderma reesei*. BMC Genomics.

[19] Damasio AR, Rubio MV, Gonçalves TA, Persinoti GF, Segato F, Prade RA (2017). Xyloglucan breakdown by endo-xyloglucanase family 74 from *Aspergillus fumigatus*. Appl Microbiol Biotechnol.

[20] Culleton H, McKie V, de Vries RP (2013). Physiological and molecular aspects of degradation of plant polysaccharides by fungi: what have we learned from *Aspergillus*?. Biotechnol J.

[21] van den Brink J, de Vries RP (2011). Fungal enzyme sets for plant polysaccharide degradation. Appl Microbiol Biotechnol.

[22] Sharma GP, Ouyang H, Wang Q, Luo Y, Shi B, Yang J (2016). Insight into enzymatic degradation of corn, wheat, and soybean cell wall cellulose using quantitative secretome analysis of *Aspergillus fumigatus*. J Proteome Res.

[23] Adav SS, Ravindran A, Sze SK (2015). Quantitative proteomic study of aspergillus fumigatus secretome revealed deamidation of secretory enzymes. J Proteome.

[24] Liu D, Li J, Zhao S, Zhang R, Wang M, Miao Y (2013). Secretome diversity and quantitative analysis of cellulolytic *Aspergillus fumigatus* Z5 in the presence of different carbon sources. Biotechnol Biofuels..

[25] Miao Y, Liu D, Li G, Li P, Xu Y, Shen Q, Zhang R (2015). Genome-wide transcriptomic analysis of a superior biomass-degrading strain of a. Fumigatus revealed active lignocellulose-degrading genes. BMC Genomics.

[26] Amore A, Giacobbe S, Faraco V (2013). Regulation of cellulase and hemicellulase gene expression in fungi. Curr Gen.

[27] Miller GL (1959). Use of dinitrosalicylic acid reagent for determination of reducing sugar. Anal Chem.

[28] Andrew S (2010). FastQC: a quality control tool for high throughput sequence data.

[29] Bolger AM, Lohse M, Usadel B (2014). Trimmomatic: a flexible trimmer for Illumina sequence data. Bioinformatics.

[30] Kopylova E, Noé L, Touzet H (2012). SortMeRNA: fast and accurate filtering of ribosomal RNAs in metatranscriptomic data. Bioinformatics.

[31] Kim D, Pertea G, Trapnell C, Pimentel H, Kelley R, Salzberg SL (2013). TopHat2: accurate alignment of transcriptomes in the presence of insertions, deletions and gene fusions. Genome Biol.

[32] Cerqueira GC, Arnaud MB, Inglis DO, Skrzypek MS, Binkley G, Simison M (2014). The aspergillus genome database: multispecies curation and incorporation of RNA-Seq data to improve structural gene annotations. Nucleic Acids Res.

[33] Nierman WC, Pain A, Anderson MJ, Wortman JR, Kim HS, Arroyo J (2005). Genomic sequence of the pathogenic and allergenic filamentous fungus *Aspergillus fumigatus*. Nature.

[34] Wang L, Wang S, Li W (2012). RSeQC: quality control of RNA-seq experiments. Bioinformatics.

[35] Liao Y, Smyth GK, Shi W (2013). The subread aligner: fast, accurate and scalable read mapping by seed-and-vote. Nucleic Acids Res.

[36] R Development Core Team (2015). R: a language and environment for statistical computing.

[37] Robinson MD, McCarthy DJ, Smyth GK (2010). edgeR: a Bioconductor package for differential expression analysis of digital gene expression data. Bioinformatics.

[38] Robinson MD, Oshlack A (2010). A scaling normalization method for differential expression analysis of RNA-seq data. Genome Biol.

[39] Glueck DH, Mandel J, Karimpour-Fard A, Hunter L, Keith E (2008). Exact calculations of average power for the Benjamini-Hochberg procedure. Int J Biostat.

[40] Hammond JBW, Kruger NJ. The Bradford method for protein quantitation. In: Walker JM, editor. New protein techniques. Methods in molecular biology™, vol 3: Humana Press; 1988. 10.1385/0-89603-126-8:25 21400151

[41] Shevchenko A, Jensen ON, Podtelejnikov AV, Sagliocco F, Wilm M, Vorm O (1996). Linking genome and proteome by mass spectrometry: large-scale identification of yeast proteins from two dimensional gels. Proc Natl Acad Sci.

[42] Pundir S, Martin MJ, O’Donovan C, UniProt Consortium. UniProt tools. Curr Protoc Bioinformatics. 2016;53:1.29–1-15. www.uniprot.org. Accessed 10 Dec 2015. 10.1002/0471250953.bi0129s53 PMC4941944 27010333

[43] Briesemeister S, Rahnenführer J, Kohlbacher O (2010). Going from where to why--interpretable prediction of protein subcellular localization. Bioinformatics.

[44] Petersen TN, Brunak S, von Heijne G, Nielsen H. SignalP 4.0: discriminating signal peptides from transmembrane regions. Nat methods. 2011;8(10):785–6. 10.1038/nmeth.1701 21959131

[45] Bendtsen JD, Jensen LJ, Blom N, von Heijne G, Brunak S (2004). Feature-based prediction of non-classical and leaderless protein secretion. Protein Eng Des Sel.

[46] Finn RD, Clements J, Arndt W, Miller BL, Wheeler TJ, Schreiber F (2015). HMMER web server: 2015 update. Nucleic Acids Res.

[47] Yin Y, Mao X, Yang JC, Chen X, Mao F, Xu Y (2012). dbCAN: a web resource for automated carbohydrate-active enzyme annotation. Nucleic Acids Res.

[48] Semighini CP, Marins M, Goldman MHS, Goldman GH (2002). Quantitative analysis of the relative transcript levels of ABC transporter Atr genes in *Aspergillus nidulans* by real-time reverse transcription-PCR assay. Appl Environ Microbiol.

[49] Ruepp A, Zollner A, Maier D, Albermann K, Hani J, Mokrejs M (2004). The FunCat, a functional annotation scheme for systematic classification of proteins from whole genomes. Nucleic Acids Res.

[50] Inglis DO, Binkley J, Skrzypek MS, Arnaud MB, Cerqueira GC, Shah P, Wymore F, Wortman JR, Sherlock G (2013). Comprehensive annotation of secondary metabolite biosynthetic genes and gene clusters of *Aspergillus nidulans*, *A. fumigatus*, *A. niger* and *A. oryzae*. BMC Microbiol.

[51] BioInforx. http://bioinforx.com/free/bxarrays/venndiagram.php Accessed 10 July 2016.

[52] Carbohydrate Active Enzymes Database. Lombard V, Golaconda Ramulu H, Drula E, Coutinho PM, Henrissat B. The carbohydrate-active enzymes database (CAZy) in 2013. Nucleic Acids Res 2014;42:D490–D495. http://www.cazy.org Accessed 26 Mar 2016. 10.1093/nar/gkt1178 PMC3965031 24270786

[53] Percival YHZ, Himmel ME, Mielenz JR (2006). Outlook for cellulase improvement: screening and selection strategies. Biotechnol Adv.

[54] Colabardini AC, Ries LN, Brown NA, Dos Reis TF, Savoldi M, Goldman MH (2014). Functional characterization of a xylose transporter in *Aspergillus nidulans*. Biotechnol Biofuels..

[55] de Souza WR, de Gouvea PF, Savoldi M, Malavazi I, Bernardes LAS, Goldman MH (2011). Transcriptome analysis of *Aspergillus niger* grown on sugarcane bagasse. Biotechnol Biofuels..

[56] Meyer V, Arentshorst M, Flitter SJ, Nitsche BM, Kwon MJ, Reynaga-Peña CG (2009). Reconstruction of signaling networks regulating fungal morphogenesis by transcriptomics. Eukaryot Cell.

[57] Lewis DA, Bisson LF (1991). The HXT1 gene product of *Saccharomyces cerevisiae* is a new member of the family of hexose transporters. Mol Cell Biol.

[58] Fan J, Chaturvedi V, Shen SH (2002). Identification and phylogenetic analysis of a glucose transporter gene family from the human pathogenic yeast *Candida albicans*. J Mol Evol.

[59] Pengli C, Ruimeng G, Bang W, Jingen L, Li W, Chaoguang T, Yanhe M (2014). Evidence of a critical role for cellodextrin transporter 2 (CDT-2) in both cellulose and hemicellulose degradation and utilization in *Neurospora crassa*. PLoS One.

[60] Tamano K, Sano M, Yamane N, Terabayashi Y, Toda T, Sunagawa M (2008). Transcriptional regulation of genes on the non-syntenic blocks of *Aspergillus oryzae* and its functional relationship to solid-state cultivation. Fungal Genet Biol.

[61] Wei H, Vienken K, Weber R, Bunting S, Requena N, Fischer R (2004). A putative high affinity hexose transporter, hxtA, of *Aspergillus nidulans* is induced in vegetative hyphae upon starvation and in ascogenous hyphae during cleistothecium formation. Fungal Genet Biol.

[62] Kwon MJ, Jørgensen TR, Nitsche BM, Arentshorst M, Park J, Ram AF, Meyer V (2012). The transcriptomic fingerprint of glucoamylase over-expression in *Aspergillus niger*. BMC Genomics.

[63] Jørgensen TR, Goosen T, Hondel CA, Ram AF, Iversen JJ (2009). Transcriptomic comparison of *Aspergillus niger* growing on two different sugars reveals coordinated regulation of the secretory pathway. BMC Genomics.

[64] Guillemette T, van Peij N, Goosen T, Lanthaler K, Robson GD, van den Hondel CA (2007). Genomic analysis of the secretion stress response in the enzyme-producing cell factory *Aspergillus niger*. BMC Genomics.

[65] Noguchi Y, Sano M, Kanamaru K, Ko T, Takeuchi M, Kato M, Kobayashi T (2009). Genes regulated by AoXlnR, the xylanolytic and cellulolytic transcriptional regulator, in *Aspergillus oryzae*. Appl Microbiol Biotechnol.

[66] Andersen MR, Nielsen J. Current status of systems biology in aspergilli. Fungal Genet Biol. 2009; 10.1016/j.fgb.2008.07.006. 10.1016/j.fgb.2008.07.006 18684401

[67] Sá-Pessoa J, Amillis S, Casal M, Diallinas G (2015). Expression and specificity profile of the major acetate transporter AcpA in *Aspergillus nidulans*. Fungal Genet Biol.

[68] Salazar M, Vongsangnak W, Panagiotou G, Andersen MR, Nielsen J (2009). Uncovering transcriptional regulation of glycerol metabolism in *Aspergilli* through genome-wide gene expression data analysis. Mol Gen Genomics.

[69] Fekete E, Karaffa L, Seiboth B, Fekete E, Kubicek CP, Flipphi M (2012). Identification of a permease gene involved in lactose utilisation in *Aspergillus nidulans*. Fungal Genet Biol.

[70] Lima MS, Damasio AR, Crnkovic PM, Pinto MR, da Silva AM, da Silva JC (2016). Co-cultivation of *Aspergillus nidulans* recombinant strains produces an enzymatic cocktail as alternative to alkaline sugarcane bagasse pretreatment. Front Microbiol.

[71] Buckeridge MS (2010). Seed cell wall storage polysaccharides: models to understand cell wall biosynthesis and degradation. Plant Physiol.

[72] Farzad S, Mandegari MA, Guo M, Haigh KF, Shah N, Görgens JF (2017). Multi-product biorefineries from lignocelluloses: a pathway to revitalisation of the sugar industry?. Biotechnol Biofuels..

[73] Calderan-Rodrigues MJ, Jamet E, Douché T, Bonassi MB, Cataldi TR, Fonseca JG (2016). Cell wall proteome of sugarcane stems: comparison of a destructive and a non-destructive extraction method showed differences in glycoside hydrolases and peroxidases. BMC Plant Biol.

[74] Buckeridge MS, de Souza AP (2014). Breaking the “Glycomic code” of cell wall polysaccharides may improve second-generation bioenergy production from biomass. Bioenergy Res.

[75] Ribeiro DA, Cota J, Alvarez TM, Bruchli F, Bragato J, Pereira BM (2012). The *Penicillium echinulatum* secretome on sugar cane bagasse. PLoS One.

[76] Saykhedkar S, Ray A, Ayoubi-Canaan P, Hartson SD, Prade RA, Mort AJ (2012). A time course analysis of the extracellular proteome of *Aspergillus nidulans* growing on sorghum Stover. Biotechnol Biofuels..

[77] Benoit I, Culleton H, Zhou M, DiFalco M, Aguilar-Osorio G, Battaglia E (2015). Closely related fungi employ diverse enzymatic strategies to degrade plant biomass. Biotechnol Biofuels.

[78] Galagan JE, Calvo SE, Borkovich KA, Selker EU, Read ND, Jaffe D (2003). The genome sequence of the filamentous fungus *Neurospora crassa*. Nature.

[79] Machida M, Asai K, Sano M, Tanaka T, Kumagai T, Terai G (2005). Genome sequencing and analysis of *Aspergillus oryzae*. Nature.

[80] Pel HJ, De Winde JH, Archer DB, Dyer PS, Hofmann G, Schaap PJ (2007). Genome sequencing and analysis of the versatile cell factory *Aspergillus niger* CBS 513.88. Nat Biotechnol.

[81] Martinez D, Berka RM, Henrissat B, Saloheimo M, Arvas M, Baker SE (2008). Genome sequencing and analysis of the biomass-degrading fungus *Trichoderma reesei* (syn. *Hypocrea jecorina*). Nat Biotechnol.

[82] Pontercorvo G, Roper JA, Hemmons LM, Macdonald KD, Bufton AWJ (1953). The genetics of *Aspergillus nidulans*. Adv Genet.

[83] Vicentini R, Bottcher A, Brito MS, Dos Santos AB, Creste S, Landell G (2015). Large-scale transcriptome analysis of two sugarcane genotypes contrasting for lignin content. PLoS One.

[84] Horta MA, Vicentini R, Delabona PS, Laborda P, Crucello A, Freitas S (2014). Transcriptome profile of *Trichoderma harzianum* IOC-3844 induced by sugarcane bagasse. PLoS One.

[85] Segato F, Damasio AR, de Lucas RC, Squina FM, Prade RA (2014). Genomics review of holocellulose deconstruction by *Aspergilli*. Microbiol Mol Biol Rev.

[86] Montanier C, van Bueren AL, Dumon C, Flint JE, Correia MA, Prates JA (2009). Evidence that family 35 carbohydrate binding modules display conserved specificity but divergent function. Proc Natl Acad Sci U S A.

[87] Dos Reis TF, de Lima PB, Parachin NS, Mingossi FB, Oliveira JVC, Ries LN, Goldman GH (2016). Identification and characterization of putative xylose and cellobiose transporters in *Aspergillus nidulans*. Biotechnol Biofuels..

[88] Turner TL, Kim H, Kong II, Liu JJ, Zhang GC, Jin YS. Engineering and evolution of *Saccharomyces cerevisiae* to produce biofuels and chemicals. Adv Biochem Eng Biotechnol. 2016; 10.1007/10_2016_22. 10.1007/10_2016_22 27913828

[89] Zhang J, Zhang B, Wang D, Gao X, Hong J (2015). Improving xylitol production at elevated temperature with engineered *Kluyveromyces marxianus* through over-expressing transporters. Bioresour Technol.

[90] Zhang W, Yi ZL, Huang JF, Li FC, Hao B, Li M (2013). Three lignocellulose features that distinctively affect biomass enzymatic digestibility under NaOH and H2SO4 pretreatments in *Miscanthus*. Bioresour Technol.

[91] Payne CM, Knott BC, Mayes HB, Hansson H, Himmel ME, Sandgren M (2015). Fungal cellulases. Chem Rev.

[92] Jordan DB, Bowman MJ, Braker JD, Dien BS, Hector RE, Lee CC (2012). Plant cell walls to ethanol. Biochem J.

[93] Ferreira ME, Colombo AL, Paulsen I, Ren Q, Wortman J, Huang J (2005). The ergosterol biosynthesis pathway, transporter genes, and azole resistance in *Aspergillus fumigatus*. Med Mycol.

[94] Hou J, Qiu C, Shen Y, Li H, Bao X. Engineering of *Saccharomyces cerevisiae* for the efficient co-utilization of glucose and xylose. FEMS Yeast Res. 2017;17:fox034. 10.1093/femsyr/fox034 28582494

[95] Berrin JG, Rosso MN, Abou HM (2017). Fungal secretomics to probe the biological functions of lytic polysaccharide monooxygenases. Carbohydr Res.

[96] Monclaro AV, Filho EXF (2017). Fungal lytic polysaccharide monooxygenases from family AA9: recent developments and application in lignocelullose breakdown. Int J Biol Macromol.

[97] Dos Santos HB, Bezerra TMS, Pradella JGC, Delabona P, Lima D, Gomes E (2016). *Myceliophthora thermophila* M77 utilizes hydrolytic and oxidative mechanisms to deconstruct biomass. AMB Express.

[98] Marx IJ, van Wyk N, Smit S, Jacobson D, Viljoen-Bloom M, Volschenk H (2013). Comparative secretome analysis of *Trichoderma asperellum* S4F8 and *Trichoderma reesei* rut C30 during solid-state fermentation on sugarcane bagasse. Biotech Biofuels.

[99] Herpoël-Gimbert I, Margeot A, Dolla A, Jan G, Mollé D, Lignon S (2008). Comparative secretome analyses of two *Trichoderma reesei* RUT-C30 and CL847 hypersecretory strains. Biotech Biofuels.

