# Supplementary material for: Transcriptome and secretome analysis of Aspergillus fumigatus in the presence of sugarcane bagasse
Source: BMC Genomics. 2018 Apr 3;19:232. doi: 10.1186/s12864-018-4627-8 (PMC5883313; doi:10.1186/s12864-018-4627-8)
Supplement: Supplementary file 1 — Figure S1. R script of RNA sequencing analysis. (PDF 2406 kb) [file 12864_2018_4627_MOESM1_ESM.pdf]

Differential Gene Expression analysis - Fructose vs Bagasse - Prof.Taisa Dinamarco USP

Diego M. Riano-Pachon

October, 13th, 2015

```
rm(list=ls())
setwd("/Users/diriano/tmp/TaisaDinamarco/")
library(edgeR)
```

```
## Loading required package: limma
```

```
library(gplots)
```

```
##
## Attaching package: 'gplots'
##
## The following object is masked from 'package:stats':
##
##     lowess
```

```
library(ggplot2)
#Import description of the experiment
targets<-read.table("targets.txt",header=T)
targets
```

```
##      Sample Treatment
## 1 frutose_1  Fructose
## 2 frutose_2  Fructose
## 3 frutose_3  Fructose
## 4     seb_1   Bagasse
## 5     seb_2   Bagasse
## 6     seb_3   Bagasse
```

```
#Import matrix of raw RNASeq counts per gene per sample
matrixCounts<-read.table("FructoseBagasse.counts.tbl",
                         header=T,row.names='Gene')
head(matrixCounts, n=3)
```

```
##            frutose_1 frutose_2 frutose_3 seb_1 seb_2 seb_3 Length
## Afu8g05820        46        16        38    63    82    24   2925
## Afu1g11050        62        40        82   216   187   208   2172
## Afu5g13370       111        72        71   155   179   148   2844
```

```
dim(matrixCounts)
```

```
## [1] 9582    7
```

```r
#Create experimental groups
GroupExp <- factor(targets$Treatment)
GroupExp
```

```
## [1] Fructose Fructose Fructose Bagasse  Bagasse  Bagasse
## Levels: Bagasse Fructose
```

```r
#Create DGE object, needed for next steps. Thi stake all
#the info for the expression analysis.
dgeExp<-DGEList(counts=matrixCounts[,1:6],
                group=GroupExp,
                genes=list(Gene=row.names(matrixCounts),
                           Length=matrixCounts[,7]))
dim(dgeExp)
```

```
## [1] 9582    6
```

```r
# Only keep genes that achieve at leat one count per million
#  in at least 3 samples, i.e., removing non-expressed genes
dgeExp_keep <- rowSums(cpm(dgeExp)>1) >= 3
dgeExp<-dgeExp[dgeExp_keep,]
dim(dgeExp)
```

```
## [1] 8578    6
```

```r
#Adjust library sizes after removing non-expressed genes
dgeExp$samples
```

```
##                group lib.size norm.factors
## frutose_1 Fructose  6927818            1
## frutose_2 Fructose  6015913            1
## frutose_3 Fructose  5708992            1
## seb_1      Bagasse  5917881            1
## seb_2      Bagasse  6239849            1
## seb_3      Bagasse  5143863            1
```

```r
dgeExp$samples$lib.size <- colSums(dgeExp$counts)
dgeExp$samples
```

```
##                group lib.size norm.factors
## frutose_1 Fructose  6925960            1
## frutose_2 Fructose  6014516            1
## frutose_3 Fructose  5707454            1
## seb_1      Bagasse  5915603            1
## seb_2      Bagasse  6237491            1
## seb_3      Bagasse  5141996            1
```

```r
#Apply TMM normalization:
# normalizes for RNA composition by finding a set of scaling factors for
# the library sizes that minimize the log-fold changes between
#the samples for most genes
dgeExp<-calcNormFactors(dgeExp)
dgeExp$samples
```

```
##               group lib.size norm.factors
## frutose_1 Fructose  6925960    0.9743033
## frutose_2 Fructose  6014516    0.8253443
## frutose_3 Fructose  5707454    0.9963732
## seb_1      Bagasse  5915603    1.1353111
## seb_2      Bagasse  6237491    1.0706010
## seb_3      Bagasse  5141996    1.0268479
```

```
#Create statistical design matrix
designExp <- model.matrix(~0+GroupExp)
colnames(designExp) <- levels(GroupExp)
designExp
```

```
##   Bagasse Fructose
## 1       0        1
## 2       0        1
## 3       0        1
## 4       1        0
## 5       1        0
## 6       1        0
## attr(,"assign")
## [1] 1 1
## attr(,"contrasts")
## attr(,"contrasts")$GroupExp
## [1] "contr.treatment"
```

```
#Compute disperssions - Estimating Biological Coefficient of Variation
#Computing Common disperssions
dgeExp<-estimateGLMCommonDisp(dgeExp,designExp)
#Computing Trended disperssions
dgeExp<-estimateGLMTrendedDisp(dgeExp,designExp)
#Computing Tagwise disperssions
dgeExp<-estimateGLMTagwiseDisp(dgeExp,designExp)

# Library size per sample.
#  The differences in library sizes were accounted for in the analysis,
#  by using the TMM normalization factors (calcNormFactors)
```

```
barplot(dgeExp$samples$lib.size*1e-6,
        names=rownames(dgeExp$samples),
        ylab="Library size (millions)",
        las=2,
        cex=0.4,
        cex.axis=1)
```

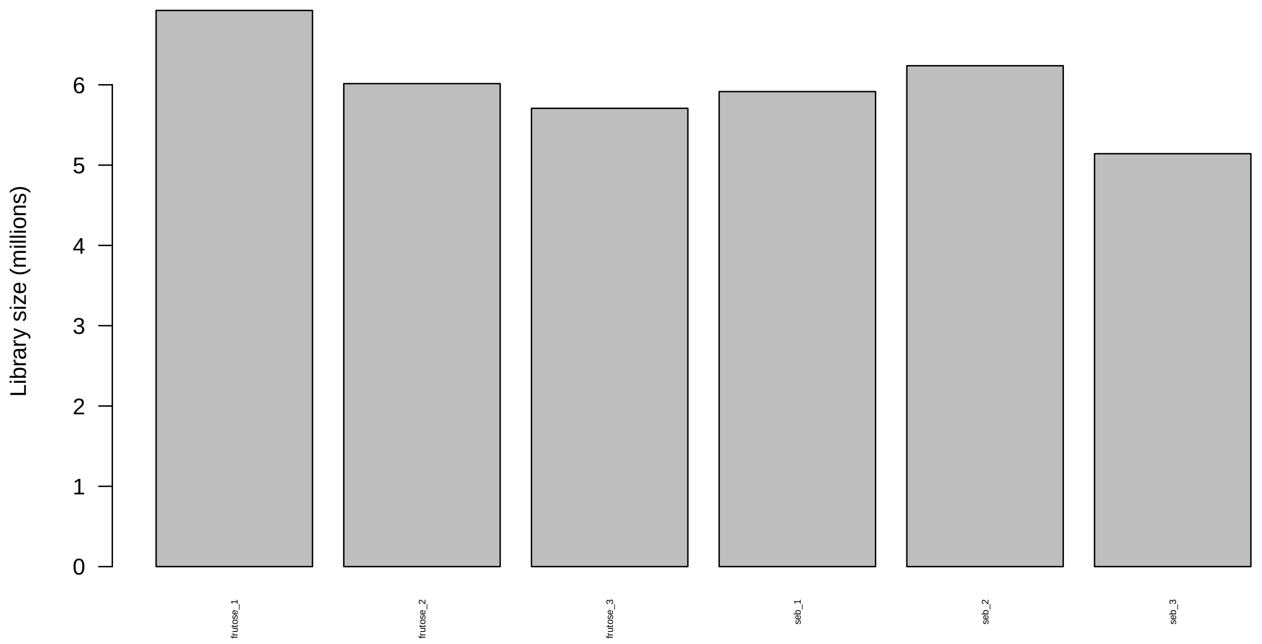


```
#Multidimissional scaling. The shows the relationships among the samples
# in gene expression space.
#From the figure it is clear that there is a clear separation of the
# samples. Dimension 2 separates WT from Mutant, while dimension 1
# separates T0 from T10
```

```
plotMDS(dgeExp,
        cex=0.5)
```

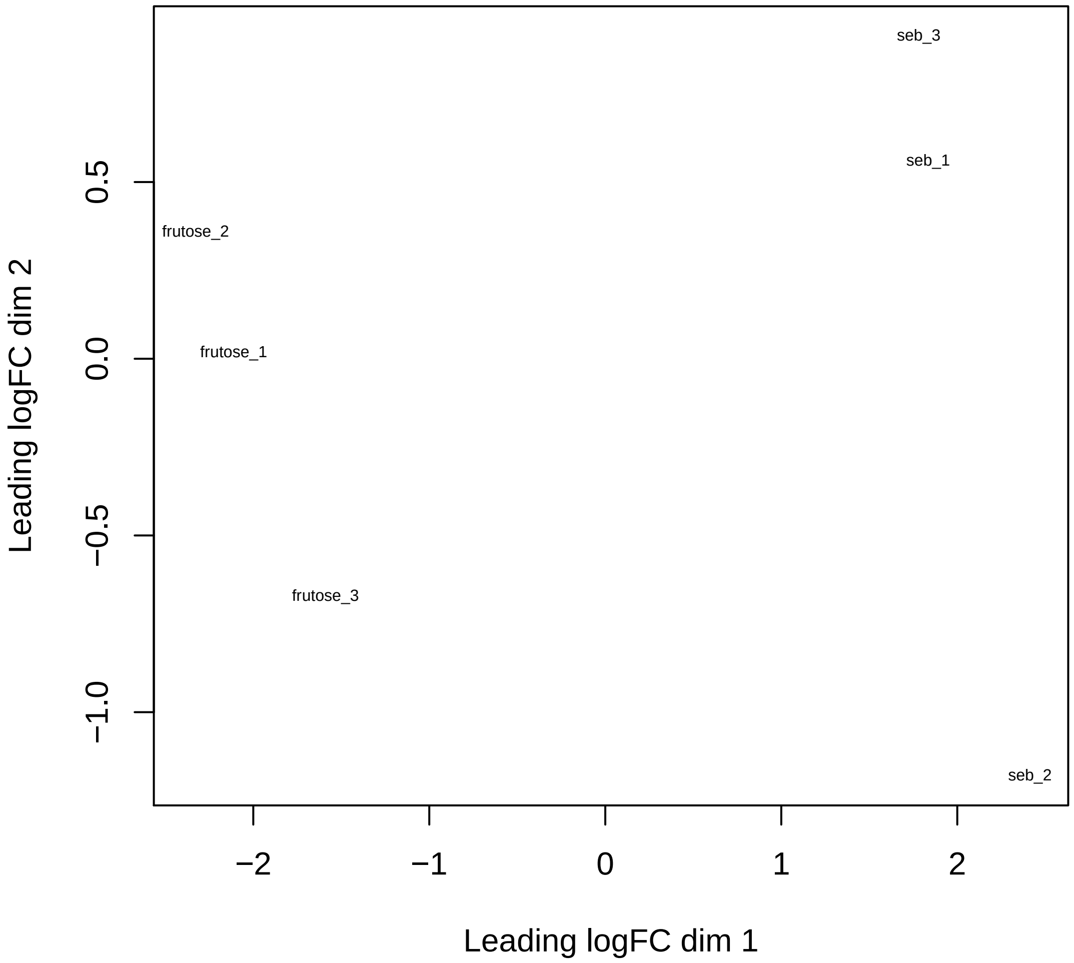


```
plotBCV(dgeExp)
```

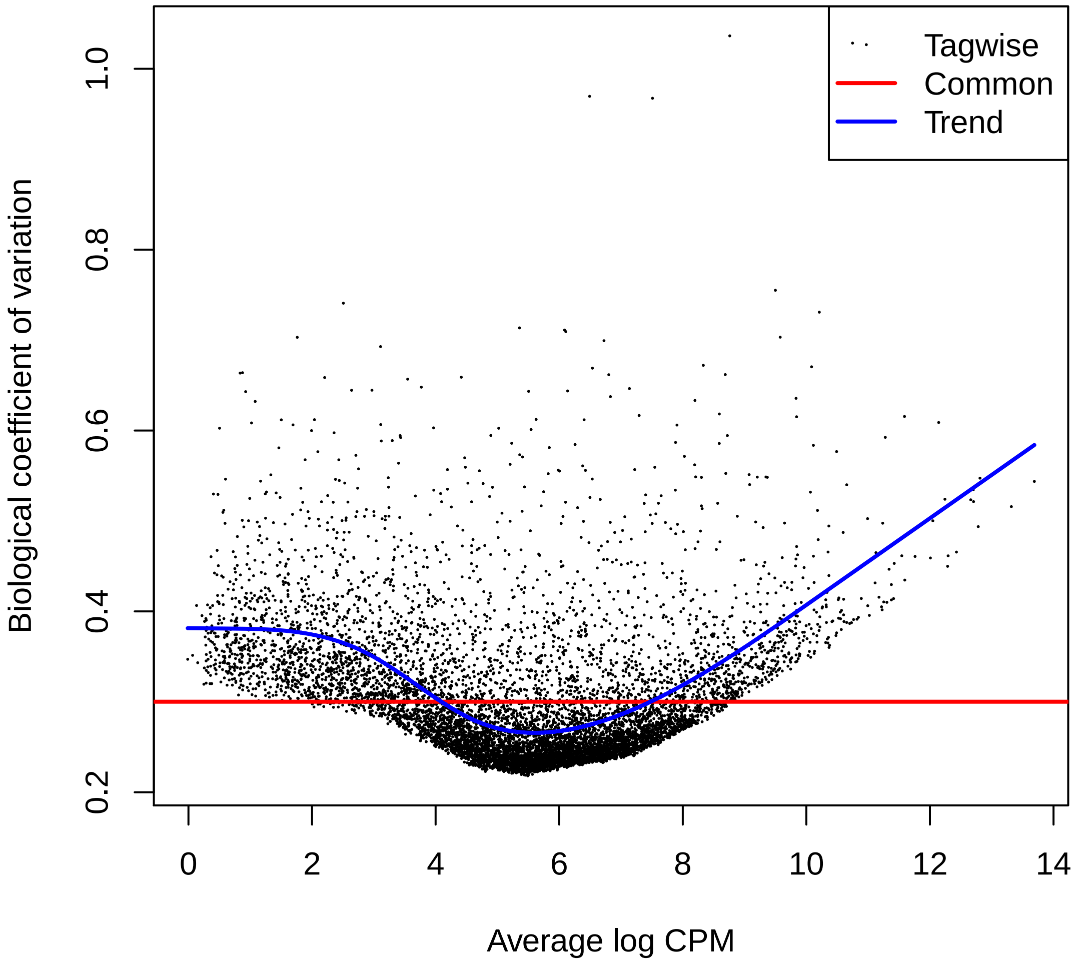


```
plotMeanVar(dgeExp,
            show.raw.vars=TRUE ,
            show.tagwise.vars=TRUE ,
            show.binned.common.disp.vars=FALSE ,
            show.ave.raw.vars=FALSE ,
            NBline = TRUE ,
            nbins = 100 ,
            pch = 16 ,
            xlab ="Mean Expression (Log10 Scale)" ,
            ylab = "Variance (Log10 Scale)" ,
            main = "Mean-Variance Plot")
```

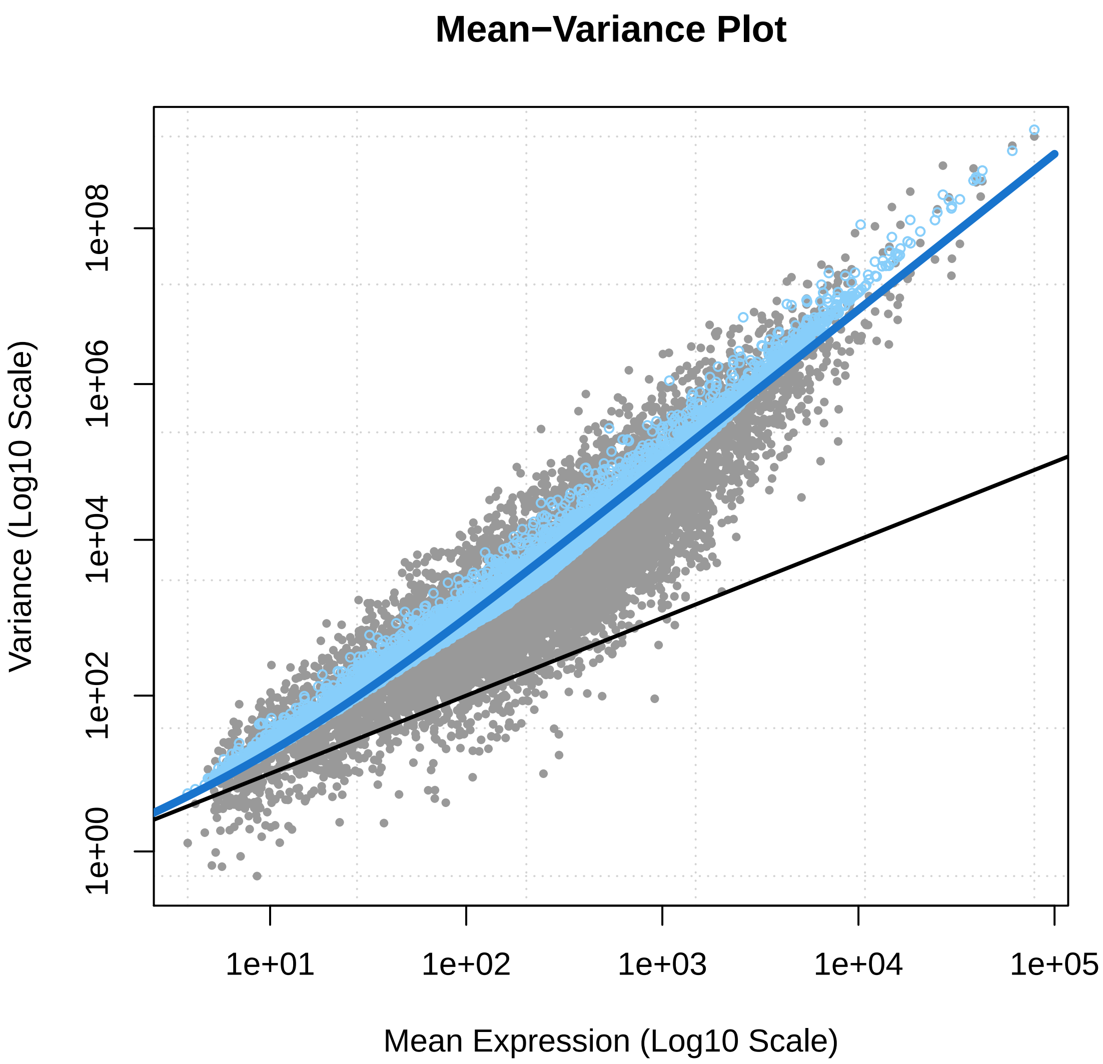


```
#Fitting the Generalized Linear Model
fitExp <- glmFit(dgeExp, designExp)
#Defining contrasts of interest
ContrastsExp<-makeContrasts(
  FvsB = Fructose-Bagasse,
  levels=designExp
)
ContrastsExp
```

```
##           Contrasts
## Levels      FvsB
##   Bagasse     -1
##   Fructose     1
```

```
#Carry out the likelihood ratio test, for the contrasts of interest
lrtFvsB <- glmLRT(fitExp, contrast=ContrastsExp[,"FvsB"])
#Flag the differentially expressed genes, correcting p-values
#using FDR/BH of 0.05
deFvsB<-decideTestsDGE(lrtFvsB)
```

```r
#Number of diferentially expressed gene in each contrast
#  And MA plot
```

deFvsB

```r
table(deFvsB)
```

```
## deFvsB
##   -1    0    1
## 1488 5716 1374
```

```r
plotSmear(lrtFvsB,
          de.tags=rownames(dgeExp)[as.logical(deFvsB)],
          ylab = 'Log2FC: Fructose vs Bagasse')
#This line (blue) represents: 1 < Log2FC < -1, or 2
#fold change up or downregulated.
abline(h=c(-1, 1),
       col="blue")
```

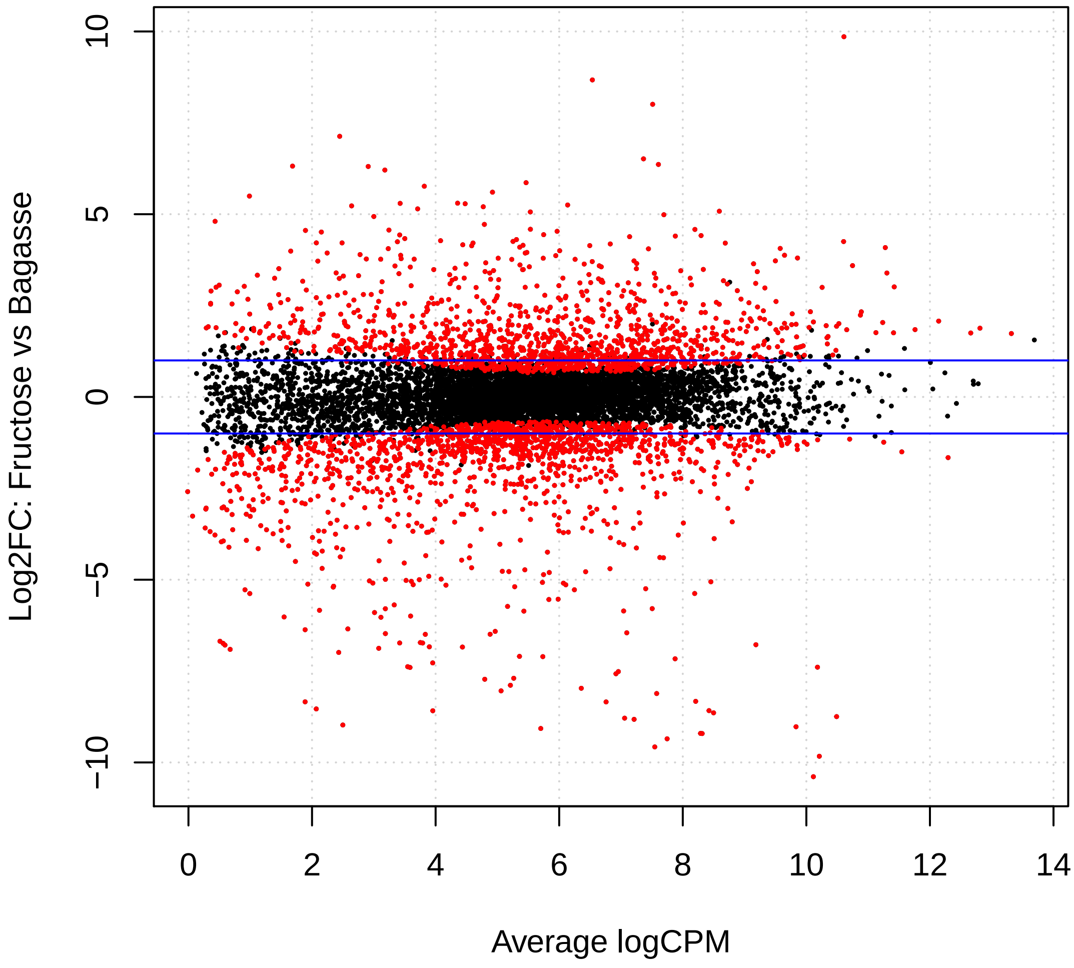

```
#Creating tables with only the data for the regulated genes
DEFvsB<-topTags(lrtFvsB,
                n=as.numeric(table(deFvsB)[3]
                             +table(deFvsB)[1]))
dim(DEFvsB)
```

```
## [1] 2862    7
```

```
#Exporting tables DE genes
write.table(DEFvsB, file="DE_Fructose_vs_Bagasse.txt",
            col.names=T,row.names=F)

#Creating matrix with FPKM values, useful for making heatmaps
matrixFPKM<-rpkm(dgeExp,normalized.lib.sizes=TRUE,
                 gene.length='Length')
head(matrixFPKM)
```

```
##             frutose_1 frutose_2 frutose_3      seb_1      seb_2      seb_3
## Afu8g05820  2.330547  1.101941  2.284511  3.2070134  4.1980770  1.5539875
## Afu1g11050  4.230169  3.709918  6.638800 14.8074417 12.8927096 18.1370092
## Afu5g13370  5.783880  5.099963  4.389998  8.1149941  9.4250975  9.8558545
## Afu6g13800  6.612258 18.153282  2.461554  0.3908059  0.5240542  0.4970925
## Afu3g04030 20.619629 15.477294 22.339939 15.3130665 21.4702898 23.4878447
## Afu2g15312  2.162562  1.701946  1.755771  3.5451679  1.6101987  4.9457264
```

```
matrixFPKM_DEG<-matrixFPKM[ which(row.names(matrixFPKM)
                                  %in% DEFvsB$table$Gene),]
dim(matrixFPKM_DEG)
```

```
## [1] 2862    6
```

```
#Exporting tables FPKM all genes
write.table(matrixFPKM, file="FructoseBagasse.FPKM.tbl",
            col.names=T,row.names=T)

#Heatmap of Z-value of FPKM for Differentially expressed genes
```

```
heatmap.2(matrixFPKM_DEG,
          scale='row',
          Colv=F,
          dendrogram='none',
          trace='none',
          density.info='none',
          margins=c(8,4))
```

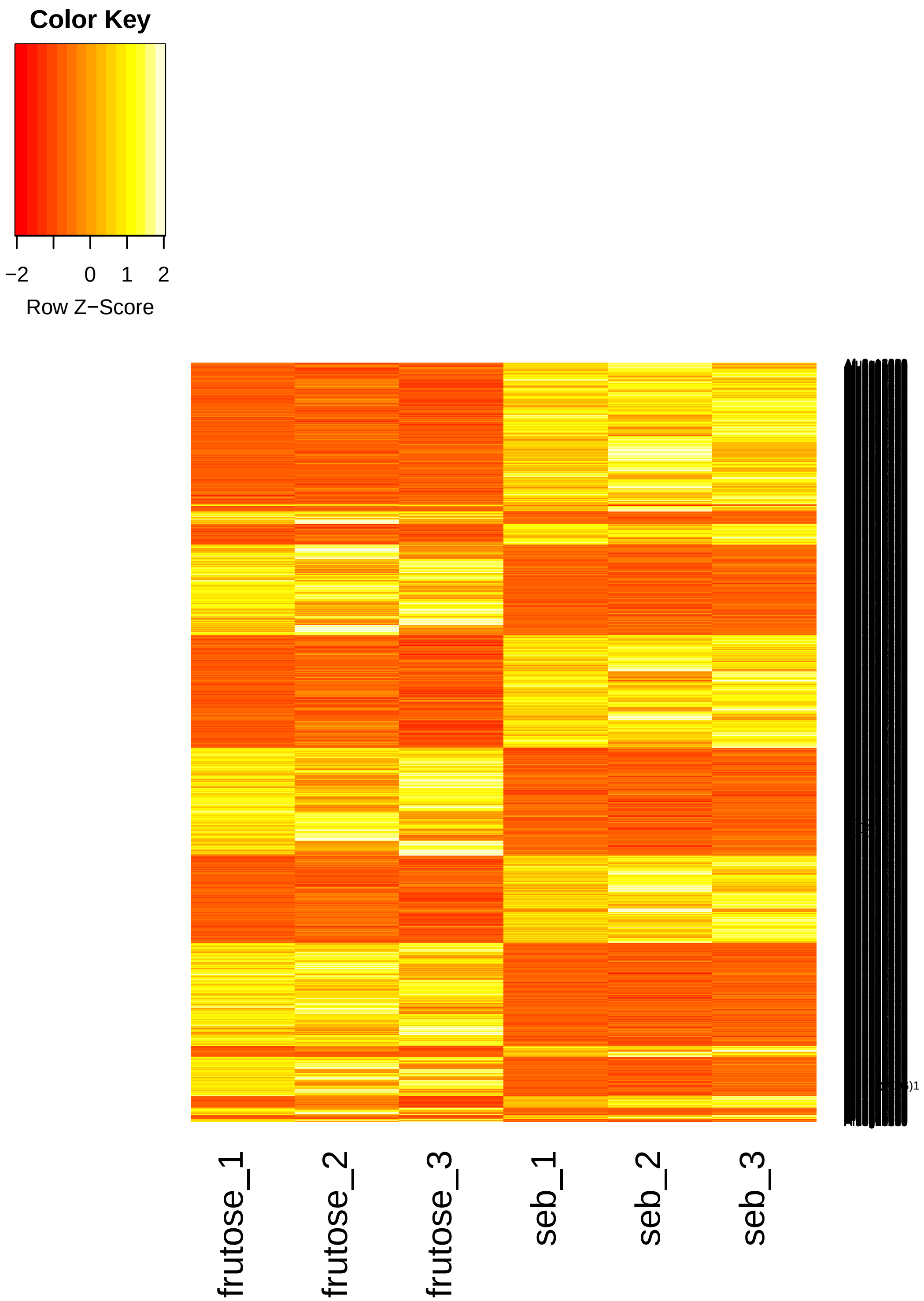


```
dbCAN<-read.delim("A_fumigatus_Af293_proteins_deducedFromGFF_dbCANv4.simple.txt",header=F)
colnames(dbCAN)<-c("Class","Family","Gene")
head(dbCAN)
```

```
##   Class Family        Gene
## 1    GH   GH18 Afu1g00310
## 2    CE    CE9 Afu1g00450
```

```
## 3    AA    AA3 Afu1g00460
## 4    AA    AA7 Afu1g00510
## 5    GH    GH3 Afu1g00540
## 6    GH   GH71 Afu1g00650
```

```r
matrixFPKM_DEG_dbCAN<-matrixFPKM_DEG[ which(row.names(matrixFPKM_DEG)
                                            %in% dbCAN$Gene),]
#Heatmap of Z-value of FPKM for dbCAN Differentially expressed genes
```

```r
heatmap.2(matrixFPKM_DEG_dbCAN,
          scale='row',
          Colv=F,
          dendrogram='none',
          trace='none',
          density.info='none',
          margins=c(8,4))
```

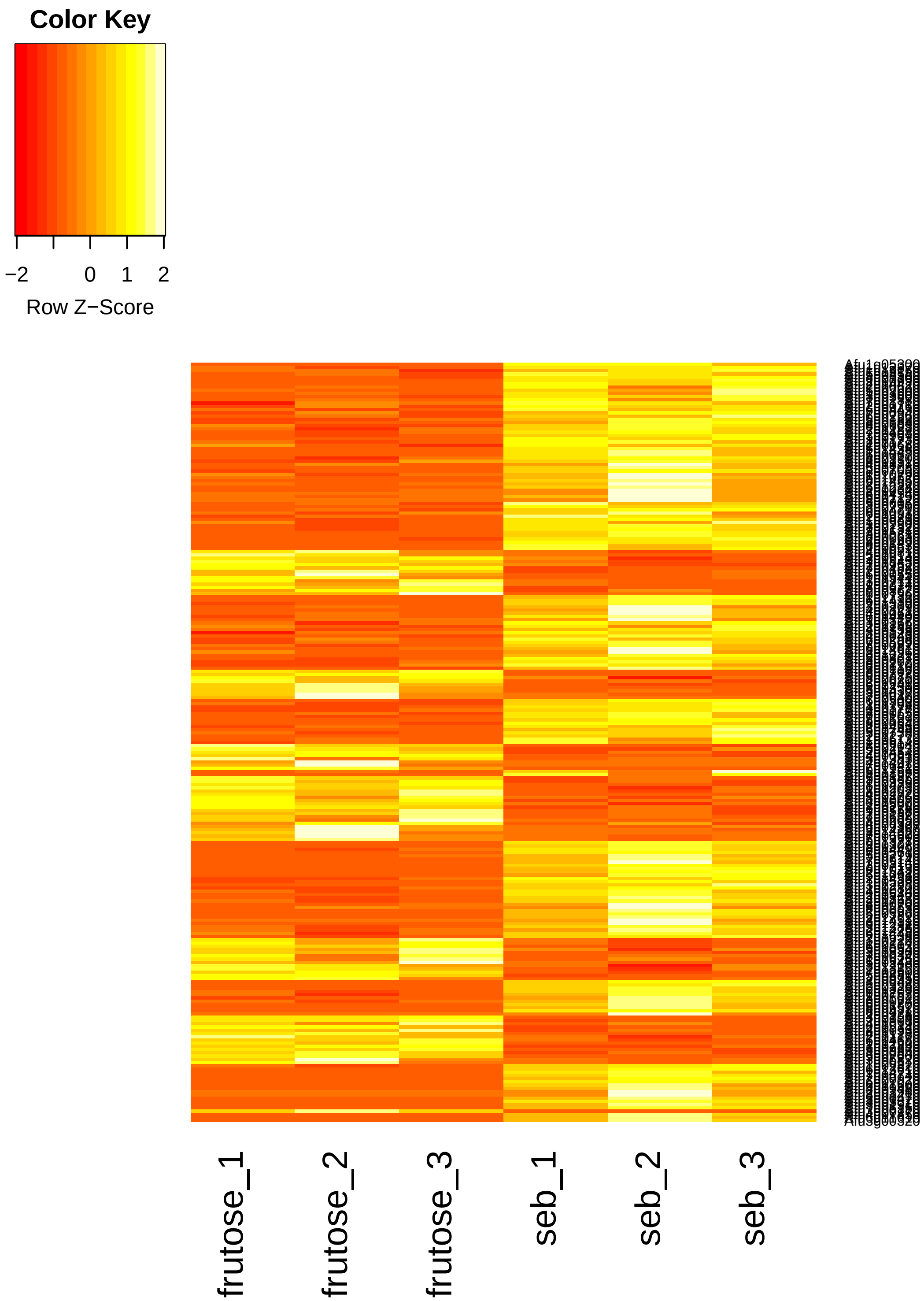


```
dbCANExpressionDGE<-data.frame(GeneID=character(),
                               dbCANClass=character(),
                               dbCANFamily=character(),
                               FructoseMean=numeric(),
                               FructoseSD=numeric(),
                               BagasseMean=numeric(),
                               BagasseSD=numeric(),
```

```
                                stringsAsFactors = FALSE
                                )

count=1
for (i in row.names(matrixFPKM_DEG_dbCAN)){
#  print(i)
  geneDE<-as.data.frame(matrixFPKM_DEG_dbCAN[i,])
  meanFructose<-mean(geneDE[c('frutose_1','frutose_2','frutose_3'),])
  sdFructose<-sd(geneDE[c('frutose_1','frutose_2','frutose_3'),])
#  print(meanFructose)
  meanBagasse<-mean(geneDE[c('seb_1','seb_2','seb_3'),])
  sdBagasse<-sd(geneDE[c('seb_1','seb_2','seb_3'),])
#  print(meanBagasse)
  dbCANDeGene<-dbCAN[which(dbCAN$Gene==i),]
#  print(dbCANDeGene)
  for (j in 1:nrow(dbCANDeGene)){
    dbCANExpressionDGE[count,'GeneID']<-i
    dbCANExpressionDGE[count,'dbCANClass']<-as.character(dbCANDeGene[j,'Class'])
    dbCANExpressionDGE[count,'dbCANFamily']<-as.character(dbCANDeGene[j,'Family'])
    dbCANExpressionDGE[count,'FructoseMean']<-meanFructose
    dbCANExpressionDGE[count,'FructoseSD']<-sdFructose
    dbCANExpressionDGE[count,'BagasseMean']<-meanBagasse
    dbCANExpressionDGE[count,'BagasseSD']<-sdBagasse
    count=count+1
  }
}

#Comparison of DE genes belonging to dbCAN families
# The diagonal represents equal expression in both treatments. We are just showing DE genes
ggplot(dbCANExpressionDGE,aes(FructoseMean,BagasseMean,colour=dbCANClass)) +
  geom_point()+
  scale_y_log10()+
  scale_x_log10()+
  geom_abline(intercept = 0, slope=1)+
  ylab('Average FPKM Bagasse') +
  xlab('Average FPKM Fructose')
```

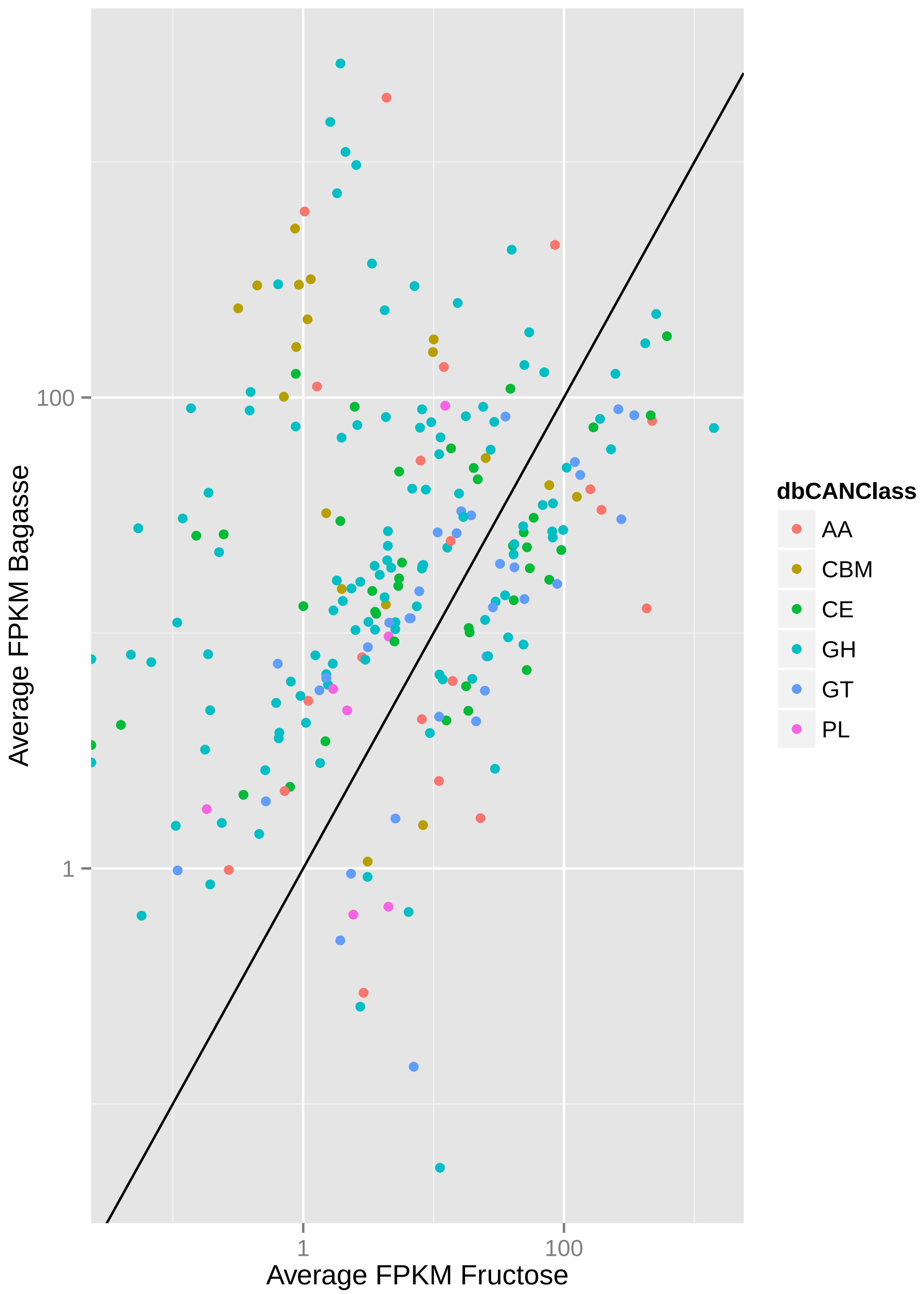


```
#Comparison of DE genes belonging to dbCAN families, same as before, but using the size paramenter,
# to represent the ratio of Bagasse to Fructose FPKM. Just to highligth the genes with largest
#  difference, over-expressed in Bagasse
# The diagonal represents equal expression in both treatments. We are just showing DE genes
ggplot(dbCANExpressionDGE,aes(FructoseMean,BagasseMean,
                              colour=dbCANClass,
```

```
                                    size=(BagasseMean/FructoseMean))) +
  geom_point()+
  scale_y_log10()+
  scale_x_log10()+
  geom_abline(intercept = 0, slope=1)+
  ylab('Average FPKM Bagasse') +
  xlab('Average FPKM Fructose')
```

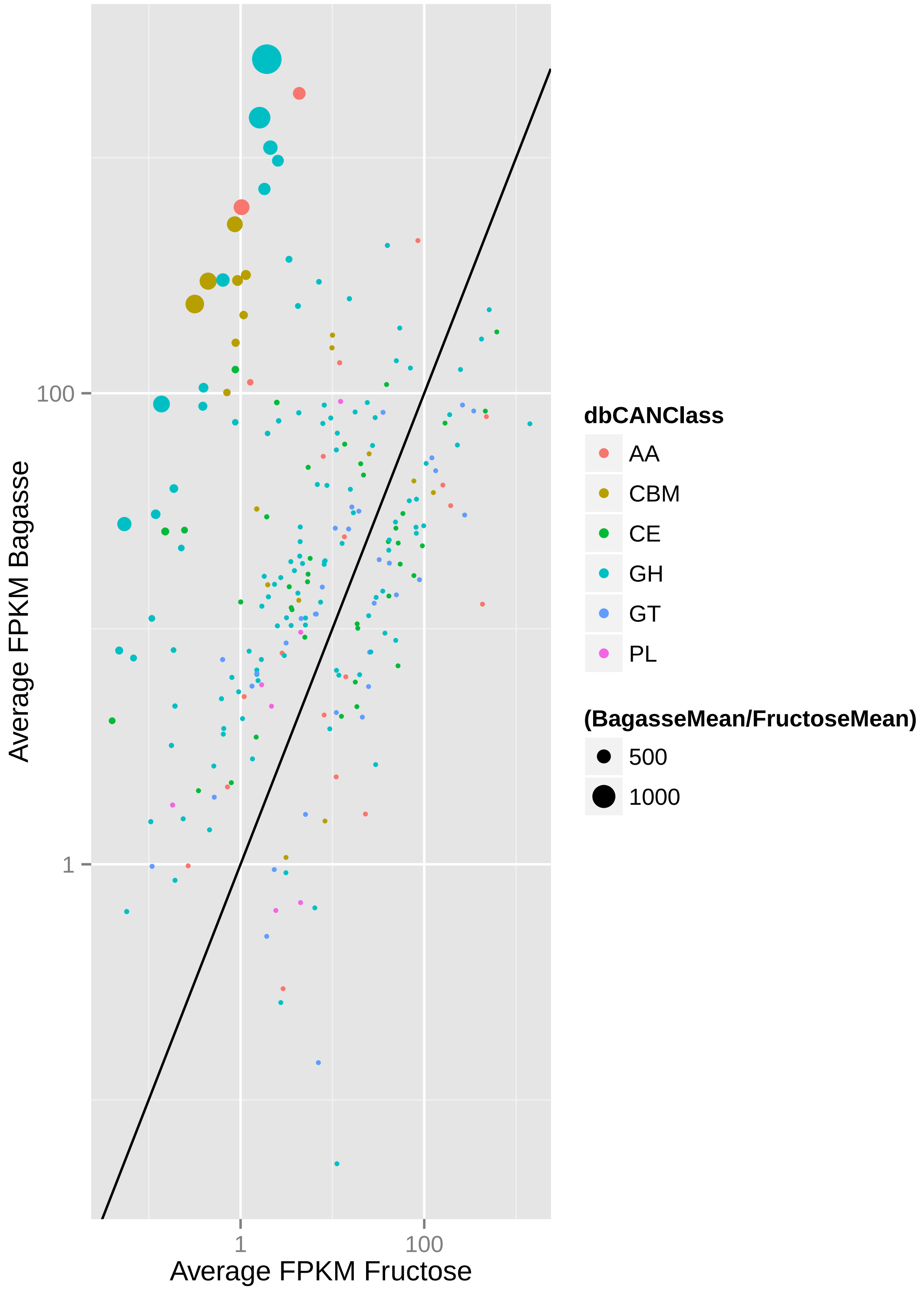


```
########
sessionInfo()
```

```
## R version 3.2.2 (2015-08-14)
## Platform: x86_64-apple-darwin13.4.0 (64-bit)
## Running under: OS X 10.10.5 (Yosemite)
```

```
##
## locale:
## [1] en_US.UTF-8/en_US.UTF-8/en_US.UTF-8/C/en_US.UTF-8/en_US.UTF-8
##
## attached base packages:
## [1] stats     graphics  grDevices utils     datasets  methods   base
##
## other attached packages:
## [1] ggplot2_1.0.1 gplots_2.17.0 edgeR_3.10.5  limma_3.24.15
##
## loaded via a namespace (and not attached):
##  [1] Rcpp_0.12.1        knitr_1.11         magrittr_1.5
##  [4] splines_3.2.2      MASS_7.3-44        munsell_0.4.2
##  [7] colorspace_1.2-6   stringr_1.0.0      plyr_1.8.3
## [10] caTools_1.17.1     tools_3.2.2        grid_3.2.2
## [13] gtable_0.1.2       KernSmooth_2.23-15 htmltools_0.2.6
## [16] gtools_3.5.0       yaml_2.1.13        digest_0.6.8
## [19] reshape2_1.4.1     formatR_1.2.1      bitops_1.0-6
## [22] evaluate_0.8       rmarkdown_0.8.1    labeling_0.3
## [25] gdata_2.17.0       stringi_0.5-5      scales_0.3.0
## [28] proto_0.3-10
```
