# Supplementary material for: Transcriptome and secretome analysis of Aspergillus fumigatus in the presence of sugarcane bagasse
Source: BMC Genomics. 2018 Apr 3;19:232. doi: 10.1186/s12864-018-4627-8 (PMC5883313; doi:10.1186/s12864-018-4627-8)
Supplement: Supplementary file 8 — Figure S2. Proteins from A. fumigatus secretome separated by SDS-PAGE. (PPTX 283 kb) [file 12864_2018_4627_MOESM8_ESM.pptx]

## Slide 1
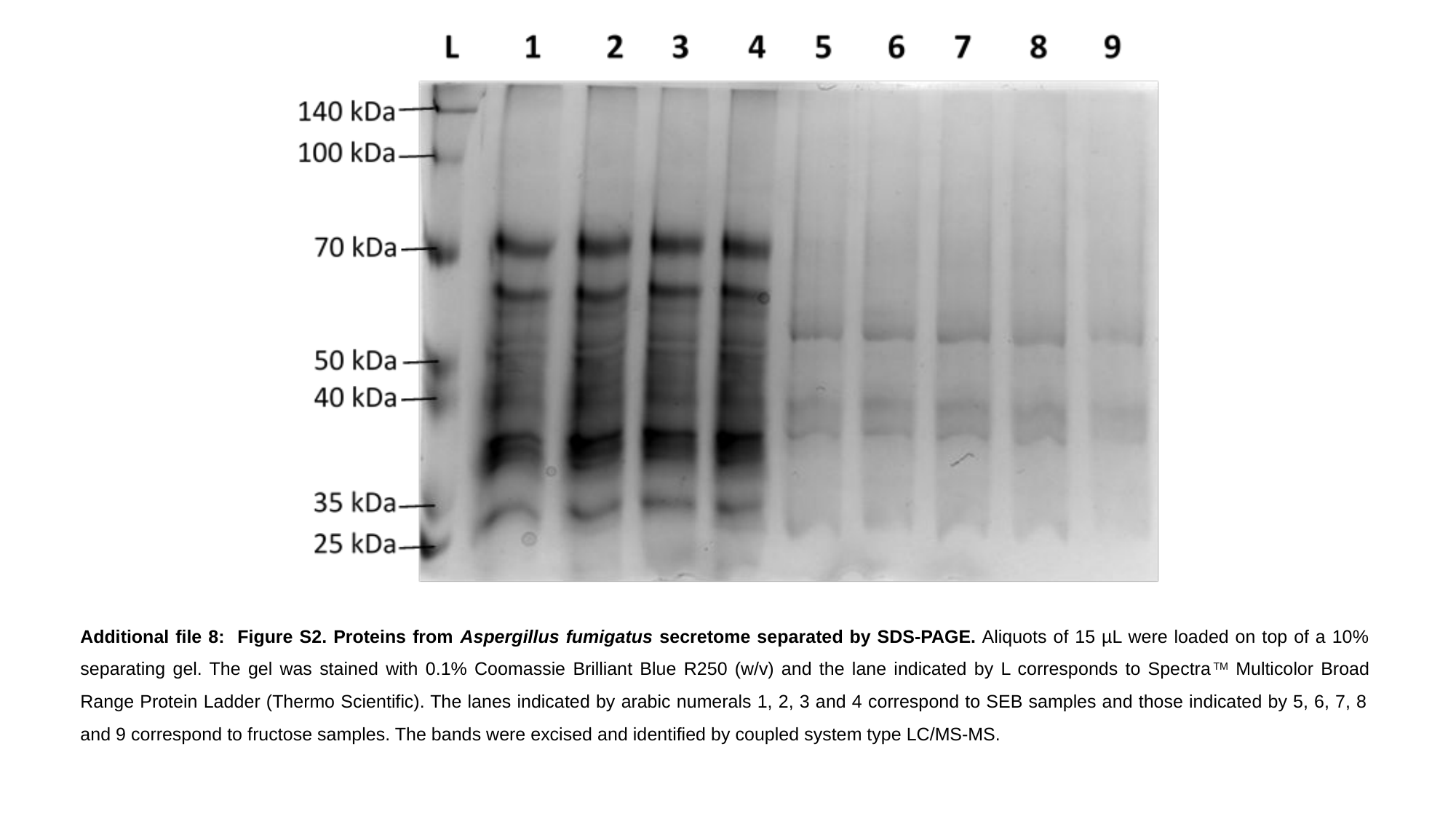

Additional file 8: Figure S2. Proteins from Aspergillus fumigatus secretome separated by SDS-PAGE. Aliquots of 15 µL were loaded on top of a 10% separating gel. The gel was stained with 0.1% Coomassie Brilliant Blue R250 (w/v) and the lane indicated by L corresponds to SpectraTM Multicolor Broad Range Protein Ladder (Thermo Scientific). The lanes indicated by arabic numerals 1, 2, 3 and 4 correspond to SEB samples and those indicated by 5, 6, 7, 8 and 9 correspond to fructose samples. The bands were excised and identified by coupled system type LC/MS-MS.
